# Flavivirus Infection Uncouples Translation Suppression from Cellular Stress Responses

**DOI:** 10.1128/mBio.02150-16

**Published:** 2017-01-10

**Authors:** Hanna Roth, Vera Magg, Fabian Uch, Pascal Mutz, Philipp Klein, Katharina Haneke, Volker Lohmann, Ralf Bartenschlager, Oliver T. Fackler, Nicolas Locker, Georg Stoecklin, Alessia Ruggieri

**Affiliations:** a Department of Infectious Diseases, Molecular Virology, University of Heidelberg, Heidelberg, Germany; b Division of Biochemistry I, Center for Biomedicine and Medical Technology Mannheim, Medical Faculty Mannheim, Heidelberg University, Mannheim, Germany; c Center for Molecular Biology of Heidelberg University (ZMBH), Heidelberg, Germany; d Division of Virus-Associated Carcinogenesis, German Cancer Research Center (DKFZ), Heidelberg, Germany; e Department of Infectious Diseases, Integrative Virology, University of Heidelberg, Heidelberg, Germany; f Faculty of Health and Medical Sciences, School of Biosciences and Medicine, University of Surrey, Guildford, United Kingdom; University of California, Irvine

## Abstract

As obligate parasites, viruses strictly depend on host cell translation for the production of new progeny, yet infected cells also synthesize antiviral proteins to limit virus infection. Modulation of host cell translation therefore represents a frequent strategy by which viruses optimize their replication and spread. Here we sought to define how host cell translation is regulated during infection of human cells with dengue virus (DENV) and Zika virus (ZIKV), two positive-strand RNA flaviviruses. Polysome profiling and analysis of *de novo* protein synthesis revealed that flavivirus infection causes potent repression of host cell translation, while synthesis of viral proteins remains efficient. Selective repression of host cell translation was mediated by the DENV polyprotein at the level of translation initiation. In addition, DENV and ZIKV infection suppressed host cell stress responses such as the formation of stress granules and phosphorylation of the translation initiation factor eIF2α (α subunit of eukaryotic initiation factor 2). Mechanistic analyses revealed that translation repression was uncoupled from the disruption of stress granule formation and eIF2α signaling. Rather, DENV infection induced p38-Mnk1 signaling that resulted in the phosphorylation of the eukaryotic translation initiation factor eIF4E and was essential for the efficient production of virus particles. Together, these results identify the uncoupling of translation suppression from the cellular stress responses as a conserved strategy by which flaviviruses ensure efficient replication in human cells.

## IMPORTANCE

For efficient production of new progeny, viruses need to balance their dependency on the host cell translation machinery with potentially adverse effects of antiviral proteins produced by the infected cell. To achieve this, many viruses evolved mechanisms to manipulate host cell translation. Here we find that infection of human cells with two major human pathogens, dengue virus (DENV) and Zika virus (ZIKV), leads to the potent repression of host cell translation initiation, while the synthesis of viral protein remains unaffected. Unlike other RNA viruses, these flaviviruses concomitantly suppress host cell stress responses, thereby uncoupling translation suppression from stress granule formation. We identified that the p38-Mnk1 cascade regulating phosphorylation of eIF4E is a target of DENV infection and plays an important role in virus production. Our results define several molecular interfaces by which flaviviruses hijack host cell translation and interfere with stress responses to optimize the production of new virus particles.

## INTRODUCTION

During infection by viruses, the translation stimulation of specific host mRNAs encoding innate response effector proteins can limit viral replication and spread (1). Therefore, interference with host mRNA translation represents a frequent evasion strategy evolved by viruses to subvert nearly every step of the host cell translation process (2). Translational arrest can be triggered by the phosphorylation of eukaryotic translation initiation factor 2 subunit α (eIF2α), which interferes with formation of the eIF2-GTP-tRNAi^Met^ ternary complex and causes stalling of translation initiation and polysome disassembly (3). Among the four eIF2α kinases, protein kinase R (PKR) is activated by viral double-stranded RNA (dsRNA) in the cytoplasm and mediates translation suppression upon replication of many RNA viruses (4). Inhibition of protein synthesis is tightly linked to the assembly of stress granules (SGs), which are cytosolic aggregates of stalled translation preinitiation complexes (5–7). As they require an intact translation machinery to translate their viral genome, several viruses antagonize SG formation during infection, although some may also exploit SG responses for their replication (8, 9).

Viruses can also interfere with host cell translation by targeting eIF4E availability or activity to limit the initial cap-binding step in the translation process (10). The activity of eIF4E is regulated by the eIF4E-binding proteins (4E-BPs), which can sequester eIF4E when hypophosphorylated, while hyperphosphorylation of 4E-BP by mechanistic target of rapamycin (mTOR) frees eIF4E (11–13). Moreover, the phosphorylation of eIF4E at serine residue 209 by the mitogen-activated protein kinase (MAPK)-interacting kinases Mnk1/2 can lead to translational activation of mRNAs encoding proteins involved with cell proliferation, inflammation, and interferon production (14–17). Therefore, several viruses manipulate mTOR or MAPK signaling pathways to exert translational control on the host (reviewed in references 2 and 10).

Dengue virus (DENV) infection is considered the most important arboviral disease (18) and causes an estimated 390 million cases annually worldwide (19). DENV infection leads to a wide spectrum of clinical manifestations ranging from asymptomatic or self-limited dengue fever to more severe symptoms, such as dengue hemorrhagic fever, dengue shock syndrome, and eventually death, which occur in a small proportion of patients and often result from secondary infections with heterologous serotypes (20–22). DENV is a member of the *Flavivirus* genus of the *Flaviviridae* family that also includes West Nile virus (WNV), Japanese encephalitis virus, yellow fever virus, and Zika virus (ZIKV). In addition to clinical manifestations similar to the febrile illness caused by DENV infection, ZIKV has recently been associated with severe neurological disease in newborns (23–25). DENV is a positive-strand RNA virus with a genome length of approximately 10.7 kb that harbors a type I cap structure at the 5′ end but lacks a polyadenylated tail at the 3′ end (26, 27). The DENV RNA genome encodes a single polyprotein that is proteolytically processed into three structural proteins (capsid, prM, and envelope) and seven nonstructural proteins (NS1, NS2A, NS2B, NS3, NS4A, NS4B, and NS5) required for viral RNA replication (28). Viral RNA is replicated through dsRNA intermediates likely shielded in virus-induced rearrangements of the endoplasmic reticulum membranes called vesicle packets (29, 30).

DENV infection interferes with cellular processes such as autophagy (31, 32) and host innate immune responses (33–35); however, the regulation of host cell translation during DENV infection is not well characterized. Paradoxically, DENV induces host stress responses but seems to repress them almost simultaneously (36–39), supposedly to avoid repression of host cell translation, which could also affect translation of the viral genome. Interestingly, DENV can switch from cap-dependent to cap-independent RNA translation when host cell translation is inhibited experimentally (40).

Herein, we aimed at defining the complex interaction of flaviviruses such as DENV and ZIKV with the host cell translation machinery in human cells. We show that flaviviruses suppress host cell translation early postinfection, while translation of their RNA genome is maintained. Translation suppression is uncoupled from the activation of the eIF2α-dependent stress response and is part of a multilayered strategy that manipulates several steps of host cell translation.

## RESULTS

### DENV induces a strong repression of host cell translation early postinfection.

**FIG 1:**
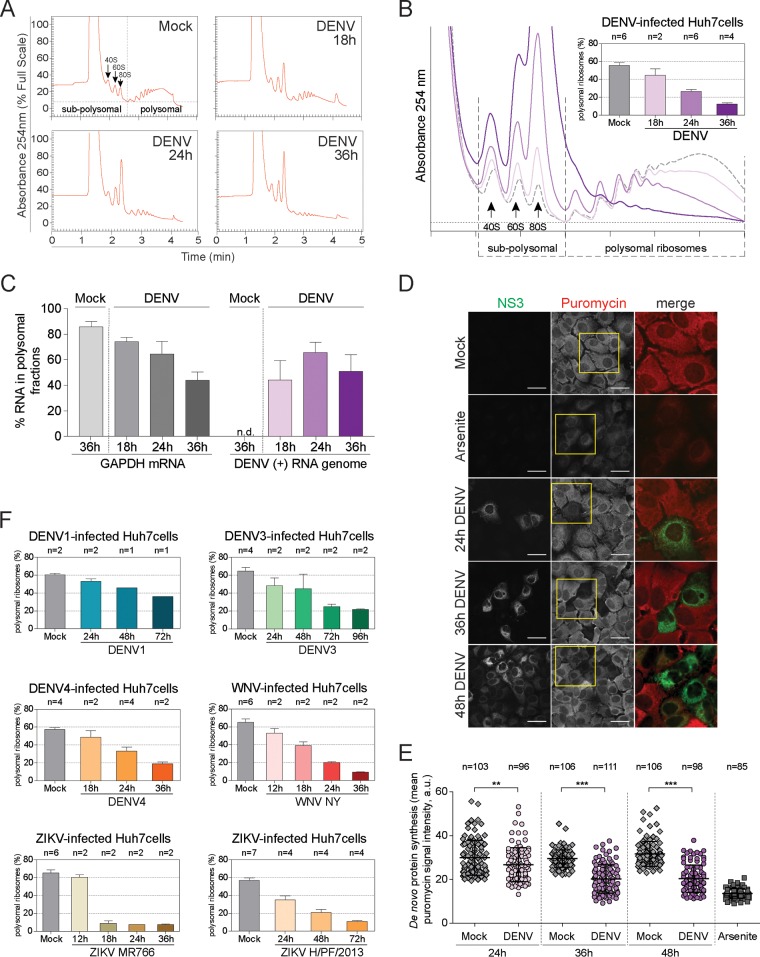
Induction of host translation repression by flavivirus infection. (A) Representative polysome profiles of naive Huh7 cells (Mock) and DENV-infected cells at 18, 24, and 36 h p.i. Cell extracts of naive Huh7 cells or cells infected with DENV (MOI of 10) for the indicated time periods were loaded on a sucrose gradient and separated by ultracentrifugation. Sucrose gradients were eluted from the top using a fractionator, and absorption at 254 nm was continuously recorded. Shown in the Mock panel is the separation between actively translated, polysomal mRNAs associated with multiple ribosomes and not or poorly translated subpolysomal mRNAs (40S and 60S, single ribosomal subunits; 80S, monosome). DENV-infected cells show an increase of the monosomal 80S peak throughout the infection. (B) Representative polysome profile analysis (lower panel). The percentage of polysomal ribosomes (actively translating mRNAs) is assessed by measuring the area below the polysomal part of the curve and the area of subpolysomal and polysomal parts of the curve. Histogram bars shown in the upper panel represent the mean percentages of polysomal ribosomes ± standard error of the mean (SEM). *n*, number of profiles analyzed. (C) Abundance of specific mRNAs in gradient fractions. Polysome profiles of naive (Mock) and DENV-infected Huh7 cells were recorded at the indicated time points p.i. Total RNA was extracted from all fractions, and the relative abundance of specific mRNAs in each fraction was quantified by qRT-PCR. Histogram bars represent mean percentages of GAPDH mRNA and DENV positive-strand RNA genome associated with the polysomal fractions ± standard deviation (SD) (*n* = 3). (D) Reduction of protein synthesis in DENV-infected cells. Naive Huh7 cells (Mock) and cells infected with DENV for the indicated time period were treated with puromycin to induce a premature release of nascent polypeptidic chains. Arsenite-treated Huh7 cells were used as a control. Puromycylated chains are visualized using an antipuromycin antibody (red) and infection by immunostaining of DENV NS3 (green). Representative fields of view are shown. Yellow squares represent the cropped section shown in the merge panel. Scale bars, 50 µm. (E) Scatter plot of *de novo* protein synthesis measured by fluorescence intensity of the puromycin signal (mean fluorescence intensities ± SD; *n* = 3) a.u., arbitrary units. Statistical significance and the number of analyzed cells (*n*) are given at the top. ***, *P* < 0.001; **, *P* < 0.01. (F) Host cell translation repression is a general feature of flavivirus infection. Polysomal profiles of Huh7 cells infected with DENV serotype 1, 3, or 4, WNV strain NY, or ZIKV MR766 or H/PF/2013 (MOI of 10) for the indicated time periods were recorded (see Fig. S2 in the supplemental material). Shown are mean percentages of polysomal ribosomes ± SEM. *n*, number of profiles analyzed.

To characterize the effect of DENV infection on global host cell translation, we compared polysome profiles from naive and DENV-infected human hepatoma Huh7 cells. DENV infections were carried out with the serotype 2 strain New Guinea C (NGC) at a high multiplicity of infection (MOI) of 10 50% tissue culture infective doses (TCID_50_) per cell, unless otherwise stated, to synchronize the infection kinetics and reduce the effects resulting from viral spread. Cell extracts were analyzed by sucrose density gradient centrifugation, and absorbance at 254 nm was continuously recorded. This allowed separating actively translated, polysomal mRNAs associated with multiple ribosomes from subpolysomal mRNAs that are not or poorly translated (Fig. 1A). As illustrated by the increase in 80S peak and concomitant decrease of the polysomal peaks, DENV-infected cells showed a progressive loss of polysomal mRNAs (Fig. 1A). This effect was already measurable 18 h postinfection (p.i.) and became even more pronounced at later times (Fig. 1A). The rate of translation for each condition was assessed by measuring the proportion of ribosomes associated with polysomes (Fig. 1B). While naive Huh7 cells had on average more than 55% polysomal ribosomes (Fig. 1B), the proportion of polysomal ribosomes strongly decreased in DENV-infected cells to 12.5% at 36 h p.i.

DENV genome translation is able to switch from a cap-dependent to a cap-independent mechanism when infected cells are treated with a translation inhibitor (40). Since the DENV positive-strand RNA genome directly binds to ribosomes for translation, we were able to investigate its association with actively translating ribosomes in the same time course experiments (Fig. 1C). Polysome profiles were recorded and fractions collected based on the elution time (see Fig. S1A in the supplemental material). Distributions of DENV positive-strand RNA genome and GAPDH (glyceraldehyde-3-phosphate dehydrogenase) mRNA, a housekeeping gene whose translation is stalled upon translation shutoff, were quantified in each fraction by quantitative reverse transcription-PCR (qRT-PCR) (Fig. S1B). As expected, the association of GAPDH mRNA with polysomal ribosomes decreased upon infection from 85 to 40% (Fig. 1C). In contrast, a constant fraction of approximately 40% of the DENV positive-strand RNA genome remained associated with polysomal ribosomes throughout the experiment, during which DENV RNA genome replication increased gradually up to 36 h p.i. (Fig. S1C). Similar translation repression of host mRNAs upon DENV infection was observed in human lung epithelial A549 cells, which are immunocompetent, in contrast to Huh7 cells (41, 42) (Fig. S1D), arguing that the observed phenotype does not depend on interferon.

10.1128/mBio.02150-16.2 Figure S1  DENV infection suppresses global protein synthesis. (A) Representative polysome profile of Huh7 cells infected with DENV (MOI of 10) for 24 h. After separation by ultracentrifugation, sucrose gradients were eluted from the top using a fractionator, and absorption at 254 nm was continuously recorded (upper panel). Fractions were collected (1 to 13), and total RNA was extracted and analyzed by formaldehyde gel electrophoresis (lower panel) to confirm the presence of 18S and 28S ribosomal RNA and distinguish precisely subpolysomal from polysomal fractions. (B) Analysis of mRNA abundance in polysome gradient fractions by qRT-PCR. After gradient fractionation, a nonhuman *in vitro* transcript (eGFP TranScript) is spiked in each fraction before purification to measure the loss of total RNA upon RNA extraction. eGFP (enhanced green fluorescent protein) transcripts are quantified in each fraction by qRT-PCR and used to normalize values of specific mRNAs. The abundance of GAPDH mRNAs in naive and DENV-infected Huh7 cells is shown as an example. Histogram bars represent the percentage of GAPDH transcripts in each fraction relative to the total amount of GAPDH transcripts in the gradient. (C) Quantification of DENV positive-strand RNA genome levels by qRT-PCR in total cell extract before separation by ultracentrifugation. All values were normalized to GAPDH mRNA levels. Shown are means ± SD from triplicate measurements from a representative experiment. (D) DENV infection induces a translational repression in human A549 cells. Shown are representative polysome profile analyses (lower panel) and mean percentages of polysomal ribosomes ± SEM (upper panel). The number of profiles analyzed (*n*) is given at the top. (E) DENV induces a reduction of global protein synthesis. Shown is representative Western blot analysis (*n* = 2) of puromycin incorporation in Huh7 cells infected with DENV for 12, 24, 36, and 48 h. Naive cells served as a control. Extracts of cells treated for 2 h with cycloheximide (CHX) were used as a control. DENV antigens were stained using DENV NS4B antiserum. GAPDH served as a loading control. Download Figure S1, PDF file, 0.4 MB. Copyright © 2017 Roth et al. 2017 Roth et al. This content is distributed under the terms of the Creative Commons Attribution 4.0 International license.

Repression of global protein synthesis in DENV-infected cells was also observed by measuring incorporation of puromycin (43), a structural tRNA analog that is covalently coupled to the carboxyl terminus of nascent polypeptides and causes their premature release from ribosomes (44). Puromycin incorporation was strongly reduced after 36 h p.i., comparable with the effect of cycloheximide (CHX), a potent translation elongation inhibitor (45) (see Fig. S1E in the supplemental material). Interestingly, viral protein levels as detected by immunostaining of DENV NS4B increased over time (Fig. S1E).

This block in cellular protein synthesis was also apparent at the single-cell level (46, 47). Huh7 cells were infected at a lower MOI (0.5 TCID_50_ per cell) to allow for parallel visualization of infected and uninfected cells and harvested at 24, 36, and 48 h p.i. Puromycylated native peptide chains were detected using an antipuromycin antibody as a measure of protein synthesis (Fig. 1D). Quantification of the puromycin signal in individual cells showed a progressive reduction of protein synthesis over the 48 h period of infection (Fig. 1E). As a positive control, cells were treated with arsenite, a potent inducer of eIF2α phosphorylation that inhibits global protein translation (48, 49). Similar protein synthesis repression was found in cells electroporated with DENV subgenomic RNA (also called a replicon [DENV_rep_]) (50), a DENV RNA genome lacking the structural protein sequences but still capable of autonomous amplification (see Fig. S2A in the supplemental material). In this system, protein suppression was stronger at later time points. This delay accounted for a slower RNA replication kinetics of the replicon (starting at 48 h postelectroporation [Fig. S2C]) compared to the replication kinetics of viral particles (Fig. S2D). We further tested the role of DENV proteins, independently of viral replication. Importantly, transient transfection of a plasmid encoding DENV nonstructural proteins NS1 to NS5 (DENV_NS1-5_) was sufficient to induce translational repression, demonstrating that viral replication is dispensable for this process (Fig. S2E and F). As expected (51), no effect on cellular translation was observed in Huh7 cells transiently expressing the hepatitis A virus replicon (HAV_rep_) or the HAV polyprotein 2ABC-3ABCD (Fig. S2B, E, and F). Together, these results establish that the DENV polyprotein potently represses host cell translation at early times p.i., irrespective of the immune competence of host cells, while RNA genome translation is maintained throughout the course of viral infection.

10.1128/mBio.02150-16.3 Figure S2  Ribopuromycylation assay. (A and B) Analysis of *de novo* protein synthesis in Huh7 cells transiently expressing DENV replicon. Huh7 cells were electroporated with wild-type DENV firefly luciferase replicon (DENV_rep_) and HAV firefly luciferase replicon (HAV_rep_) as a control. Cells were treated with puromycin at the indicated time points. After fixation, puromycylated polypeptidic chains were visualized using an antipuromycin antibody. Viral antigens were immunostained with DENV NS3 antiserum and HAV proteinase 3C antiserum. Shown are scatter plots of puromycin mean fluorescence intensities (a.u.) ± SD from a representative experiment. Statistical significance and the number of analyzed cells (*n*) are given at the top. ***, *P* < 0.001; n.s., not significant. (A) Huh7 cells expressing DENV_rep_ (*n* = 3). (B) Huh7 cells expressing HAV_rep_ (*n* = 2). (C and D) Analysis of DENV firefly luciferase replicons and DENV serotype 2 strain NGC replication kinetics. (C) The DENV replicon system expresses a firefly luciferase reporter gene that allows for the measurement of luciferase activity as a surrogate of RNA replication. *In vitro* transcripts of wild-type DENV firefly luciferase replicon (DENV_rep_) and replication-defective DENV firefly luciferase replicon (DENV_rep_ GND) were electroporated in Huh7 cells and harvested at 4, 24, 48, and 72 h postelectroporation. To assess DENV_rep_ RNA replication, cells were lysed at the time points specified, and firefly luciferase activities were determined (relative light units [RLU]). Values were normalized to the 4 h (input RNA) value. Shown are mean RLU values ± SD from three independent experiments. (D) Huh7 cells (1 × 10^5^) were infected at an MOI of 0.1 TCID_50_ per cell for 2 h. Twenty-four, 48, 72, and 96 h postinfection, cells were harvested, and infectious titers were determined by limiting dilution assay (TCID_50_ per milliliter). Shown are mean values ± SD from three independent experiments. (E and F) DENV polyprotein is sufficient for translational repression. Expression of DENV polyproteins NS1 to NS5 and HAV polyprotein in Huh7 Lunet T7 cells. Forty-eight hours posttransfection, cells were treated with puromycin and fixed. (E) Representative fields of view are shown. Yellow squares represent the cropped section shown in the merge panel. Scale bars, 50 µm. (F) Scatter plots of puromycin mean fluorescence intensities ± SD from a representative experiment (*n* = 3). a.u., arbitrary units. Statistical significance and the number of analyzed cells (*n*) are given at the top. ***, *P* < 0.001; n.s., not significant. Download Figure S2, PDF file, 0.2 MB. Copyright © 2017 Roth et al. 2017 Roth et al. This content is distributed under the terms of the Creative Commons Attribution 4.0 International license.

### Host cell translation repression is a hallmark of flavivirus infection.

To test whether early repression of host cell translation is a general feature of flavivirus infection, we analyzed other DENV serotypes (DENV1, DENV3, and DENV4), ZIKV Uganda strain MR766 and Polynesian strain H/PF/2013, as well as WNV strain New-York 99 (here, WNV NY). Infection kinetics were defined based on virus replication and cytopathogenicity in Huh7 cells. Of note, cell confluence was maintained at a maximum of 95%, even at late time points after infection to avoid translation repression artifacts due to growth restriction. As shown by polysome profile analyses (see Fig. S3A to F in the supplemental material) and estimation of translation rates (Fig. 1F), all flaviviruses analyzed repressed host cell translation, albeit to different extents. DENV4 and WNV NY infection induced a repression comparable to that observed with DENV2 NGC infection. Infection with DENV1, DENV3, and ZIKV H/PF/2013 induced a slower and slightly less potent translation repression, which correlated with a reduced cytopathic effect in Huh7 cells compared to DENV2 NGC. Finally, infection with ZIKV strain MR766 induced a very strong reduction of translation rates as early as 18 h p.i. The suppression of a global translation accompanied by the disassembly of heavy polysomes is therefore a general feature of flavivirus infection.

10.1128/mBio.02150-16.4 Figure S3  Polysome profiles of Huh7 cells infected with flaviviruses. Huh7 cells were infected (MOI of 10) with (A) DENV serotype 1 strain Hawaii (DENV1), (B) DENV serotype 3 strain H87 (DENV3), (C) DENV serotype 4 strain H241 (DENV4), (D) WNV strain New-York 99 (WNV NY), (E) ZIKV strain MR766, or (F) ZIKV strain H/PF/2013. Shown are representative polysome profile analyses (lower panels) and mean percentages of polysomal ribosomes ± SEM (upper panels). The number of profiles analyzed (*n*) is given at the top. Download Figure S3, PDF file, 0.3 MB. Copyright © 2017 Roth et al. 2017 Roth et al. This content is distributed under the terms of the Creative Commons Attribution 4.0 International license.

### Host cell translation is impaired at the initiation stage.

**FIG 2:**
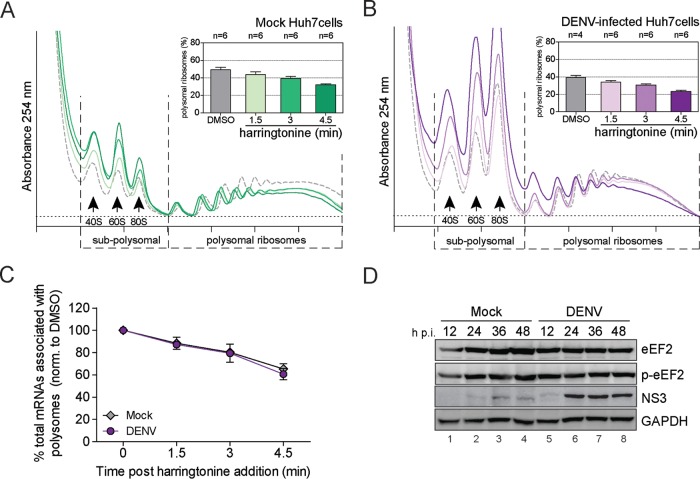
Host cell translation is impaired at the initiation step by DENV infection. (A, B, and C) DENV infection does not affect RNA translation elongation. Naive Huh7 cells (Mock [A]) and cells infected with DENV (MOI of 10) for 24 h (B) were treated with harringtonine for 1.5, 3, and 4.5 min to allow ribosome runoff. Treatment with DMSO for 4.5 min was used as a control. Shown are representative polysome profile analyses (lower panel) and mean percentages of polysomal ribosomes ± SEM (upper panel; *n*, number of profiles analyzed). (C) Mean percentages ± SD (*n* = 3) of total mRNAs associated with polysomes in mock- and DENV-infected cells upon harringtonine treatment (corresponding to panels A and B) were normalized to the values of DMSO-treated cells, respectively. (D) Phosphorylation levels of the eEF2 are not affected by DENV infection. Shown is representative Western blot analysis (*n* = 2) of Huh7 cells infected with DENV (MOI of 10) for 12, 24, 36, and 48 h (lanes 5 to 8). Naive Huh7 cells (Mock) cultured in parallel for the same time periods were used as reference (lanes 1 to 4).

Global protein synthesis shutoff can result from reducing the rate of translation initiation or elongation (52). Our previous observation that subpolysomal mRNAs are markedly increased in DENV-infected cells (Fig. 1B) suggested repression of translation at the initiation stage. To test if elongation was also affected, we analyzed translation elongation rates by ribosome runoff experiments (53). Naive Huh7 cells (Fig. 2A) or Huh7 cells infected with DENV for 24 h (Fig. 2B) were treated with harringtonine, an alkaloid that inhibits translation initiation only by a block following 60S subunit joining (54). Polysome profile analysis revealed a similar decrease of polysomes due to ribosome runoff over the 4.5-min treatment in naive and DENV-infected cells (Fig. 2C), indicating similar rates of translation. Consistently, phosphorylation levels of eukaryotic elongation factor 2 (eEF2), reflecting altered binding to the ribosome and impaired elongation (55), remained unaltered during the course of infection (Fig. 2D). We conclude that DENV infection impairs host cell translation at the initiation step but does not alter translation elongation.

### DENV-induced host cell translation initiation repression is uncoupled from host cell stress responses.

**FIG 3:**
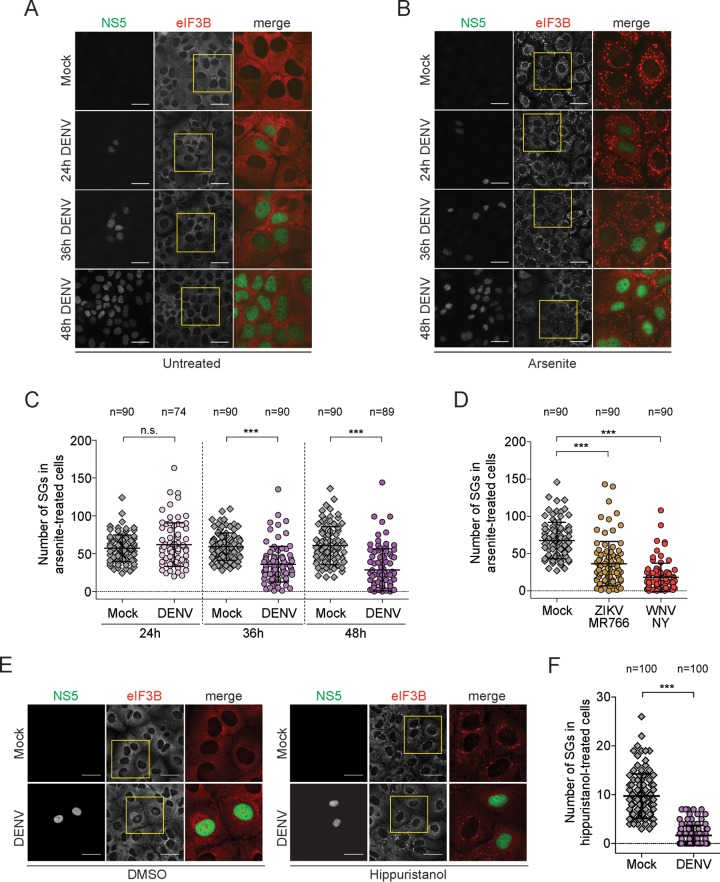
Flavivirus infection inhibits eIF2α-dependent and -independent SG formation in Huh7 cells (A, B, and C). Huh7 cells were infected with DENV (MOI of 0.5) for the indicated time period and left untreated (A) or treated with arsenite (B) before fixation. DENV infection was visualized by immunostaining of NS5 (green) and SGs by immunostaining of eIF3B (red). Naive cells (Mock) served as control. Representative fields of view are shown. Yellow squares represent the cropped section shown in the merge panel. Scale bar, 50 µm. (C) Scatter plot displaying the number of SGs in cells treated with arsenite. Shown are mean values ± SD of a representative experiment (*n* = 3). *n*, number of cells analyzed. ***, *P* < 0.001; n.s., not significant. (D) ZIKV and WNV inhibit arsenite-induced SG assembly. Similar to panel C, Huh7 cells were infected with ZIKV MR766 or WNV NY (MOI of 0.5) for 24 h and treated with arsenite before fixation. (E and F) Huh7 cells were infected with DENV (MOI of 0.5) for 30 h and treated with hippuristanol for 8 h before fixation. (E) DENV infection was visualized by immunostaining of NS5 (green) and SGs by immunostaining of eIF3B (red). Naive cells (Mock) served as control. Representative fields of view are shown. Yellow squares represent the cropped section shown in the merge panel. Scale bars, 50 µm. (F) Scatter plot displaying the number of SGs in cells treated with hippuristanol. Shown are mean values ± SD from a representative experiment (*n* = 3). *n*, number of cells analyzed. ***, *P* < 0.001.

Stalling of translation initiation is tightly linked to SG assembly (5). To overcome host translation shutoff, several RNA viruses evolved different strategies, including mechanisms that interfere with SG formation (8, 9). It has been reported that DENV inhibits SG formation in baby hamster kidney (BHK-21) cells by sequestering the SG-initiating proteins T cell internal antigen-1 (TIA-1) and TIA-1-related protein R (TIAR) on the 3′ untranslated region (UTR) of its RNA genome (36), although we found previously that infection of Huh7 cells with DENV induced few sporadically oscillating SGs (56). To test if DENV-induced translation initiation repression was linked to the activation of a (sporadic) stress response in Huh7 cells, we analyzed SG formation in detail (Fig. 3A). The eukaryotic initiation factor eIF3B, which binds the 40S subunit, was used as bona fide SG marker. No SGs were detectable in DENV-infected Huh7 cells (Fig. 3A), and as expected (36), in WNV NY-infected cells (see Fig. S4A in the supplemental material). Next, we assessed the response to arsenite-induced oxidative stress, which causes translation suppression and SG formation through eIF2α phosphorylation. When treated with arsenite, DENV-infected cells showed an attenuation of SG formation affecting both the number (Fig. 3B and C) and size (Fig. S4C) of SGs. Moreover, inhibition of arsenite-induced SG formation in DENV-infected cells correlated negatively with the viral expression level, as measured by the accumulation of NS5 signal intensity in the nucleus (Fig. S4D). In agreement with earlier reports (36, 57), these results demonstrate that DENV and WNV NY (Fig. 3D) repress eIF2α phosphorylation-dependent SG formation in Huh7 cells.

10.1128/mBio.02150-16.5 Figure S4  Inhibition of arsenite-induced SG formation by DENV and ZIKV infection. (A and B) ZIKV and WNV inhibit SG assembly. Naive Huh7 cells and Huh7 cells infected with ZIKV MR766 and WNV NY (MOI of 0.5) for 24 h were left untreated (A) or treated with arsenite for 45 min (B) before fixation. ZIKV and WNV infection was visualized by immunostaining of dsRNA (green) and SGs by immunostaining of eIF3B (red). Naive cells (Mock) served as a control. Representative fields of view are shown. Yellow squares represent a cropped section shown in the merge panel. Scale bars, 50 µm. (C and D) Inhibition of arsenite-induced SGs by DENV infection. Huh7 cells were infected with DENV (MOI of 0.5) for the indicated time periods and left untreated or treated with 500 μM arsenite for 45 min before fixation. DENV infection was visualized by immunostaining of NS5 and SGs by immunostaining of eIF3B (Fig. 3). (C) Scatter plot of SG size (square micrometers) in cells treated with arsenite. Shown are mean values ± SD from a representative experiment (*n* = 3). Statistical significance and the number of analyzed cells (*n*) are given at the top. ***, *P* < 0.001; **, *P* < 0.01. (D) Scatter plot of correlation between DENV NS5 mean fluorescence intensity (reflecting the level of DENV replication) and number of arsenite-induced SGs in DENV-infected cells at 24, 36, and 48 h postinfection. *n*, number of analyzed cells; PC, Pearson’s correlation; *R*^2^, coefficient of determination. (E) SG-like foci in ZIKV-infected cells. Huh7 cells were infected with ZIKV MR766 for 24 h. ZIKV infection was visualized by immunostaining of dsRNA (green) or NS3 (far red) and SGs by immunostaining of eIF3B (red), HuR, and PCBP2 (green), and TIAR (red) and analyzed by confocal microscopy. Representative fields of view are shown. Yellow squares represent the cropped section shown in the merge panel. Scale bar, 50 µm. (F) Treatment with hippuristanol does not induce eIF2α phosphorylation. Naive Huh7 cells infected with DENV (MOI of 10) for 30 h were left untreated or treated with arsenite, an eIF2α kinase-inducing stressor, and hippuristanol, an eIF2α kinase-independent stressor. Shown is representative Western blot analysis of phospho-eIF2α (p-eIF2α) abundance (*n* = 2). Download Figure S4, PDF file, 1.4 MB. Copyright © 2017 Roth et al. 2017 Roth et al. This content is distributed under the terms of the Creative Commons Attribution 4.0 International license.

Surprisingly, although infection with ZIKV strain MR766 was also capable of repressing arsenite-induced SG formation to levels similar to those of DENV infection (Fig. 3D; see also Fig. S4B in the supplemental material), small eIF3B positive-foci, which colocalized with the SG markers Hu protein R (HuR), poly(rC)-binding protein 2 (PCBP2) and TIAR, were detected in around 55% of naive Huh7 cells infected with ZIKV (Fig. S4A and E).

SG formation can be triggered in an eIF2α phosphorylation-independent manner by treatment with hippuristanol (58), an inhibitor of eIF4A RNA binding (59). Huh7 cells infected with DENV were treated with hippuristanol and compared to dimethyl sulfoxide (DMSO)-treated cells (Fig. 3E). Remarkably, DENV infection also impaired hippuristanol-induced SG formation (Fig. 3F) in an eIF2α phosphorylation-independent manner (see Fig. S4E in the supplemental material). Altogether, these results suggest that DENV infection inhibits SG formation induced by both eIF2α-dependent and -independent pathways.

### DENV-induced repression of host cell translation is independent of PKR and eIF2α phosphorylation.

**FIG 4:**
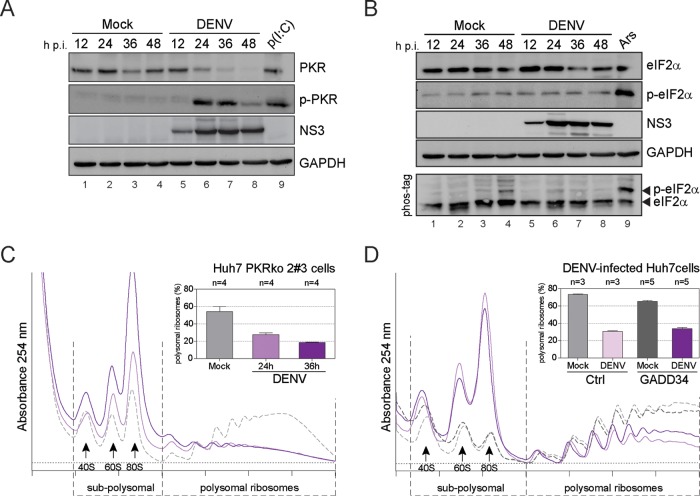
DENV-induced host cell translation repression is independent of the PKR-eIF2α signaling pathway. (A and B) activation of PKR by DENV does not result in eIF2α phosphorylation. Cells were infected with DENV (MOI of 10) for 12, 24, 36, and 48 h (lanes 5 to 8). Naive Huh7 cells (Mock) cultured in parallel for the same time periods were used as reference (lanes 1 to 4). Shown are representative Western blot analyses (*n* = 4). (A) Analysis of PKR and phospho-PKR (p-PKR) abundance. Cells transfected with the synthetic dsRNA poly(I-C) (lane 9) were used as a positive control. (B) Analysis of eIF2α and phospho-eIF2α (p-eIF2α) abundance. Cells treated with arsenite (lane 9) were used as a positive control. Phosphorylation of eIF2α was analyzed by Phos-tag acrylamide gel (lower panel). (C) DENV-induced translational repression is PKR independent. Polysome profiles of Huh7 PKR ko cells (clone 2#3) left untreated (Mock) or infected with DENV (MOI of 10) were recorded at the indicated times. Shown are representative polysome profile analyses (lower panel) and mean percentages of polysomal ribosomes ± SEM (upper panel). *n*, number of profiles analyzed. (D) DENV-induced translational repression is eIF2α independent. Polysome profiles of Huh7 control cells (Ctrl) and Huh7 cells stably expressing GADD34 and infected with DENV (MOI of 10) were recorded 24 h p.i. Naive cells (Mock) were used as a control. Shown are representative polysome profile analyses (lower panel) and mean percentages of polysomal ribosomes ± SEM (upper panel). *n*, number of profiles analyzed.

Cells respond to various stressors, including viral infection by inducing phosphorylation of eIF2α, which results in translational stalling. Since inconsistent results have been observed in different experimental systems regarding the phosphorylation status of eIF2α in the course of DENV infection (37, 38), we tested whether the PKR-eIF2α signaling pathway is activated by DENV infection in Huh7 cells despite the absence of SGs. Remarkably, and in agreement with previous reports (35), basal PKR levels were dramatically reduced in the course of DENV infection. However, PKR phosphorylation was markedly increased at 24 h p.i., a time at which SGs were not detected (Fig. 4A). In stark contrast, eIF2α phosphorylation remained at basal levels in DENV-infected cells at all time points examined (Fig. 4B). Similarly, the analysis of global eIF2α phosphorylation by Phos-tag acrylamide gel electrophoresis (60), a mobility shift detection assay of phosphorylated proteins, confirmed the absence of eIF2α phosphorylation (Fig. 4B, lower panel). Of note, impairment of eIF2α phosphorylation in response to kinases other than PKR was previously reported (36, 37). Consistently, DENV and ZIKV infection blocked eIF2α phosphorylation induced by treatment with arsenite, which activates both heme-regulated eIF2α kinase (HRI) and general control nonderepressible 2 (GCN2), thapsigargin, which activates the PKR-like endoplasmic reticulum kinase (PERK), and carbonyl cyanide *p*-(trifluoromethoxy) phenylhydrazone (FCCP), which activates HRI (61) (see Fig. S5A to F in the supplemental material).

10.1128/mBio.02150-16.6 Figure S5  Flaviviruses impair eIF2α phosphorylation by other eIF2α kinases. (A, B, and C) Naive Huh7 cells and Huh7 cells infected with DENV (MOI of 10) for 30 h were left untreated or treated with different eIF2α kinase-inducing stressors, including arsenite, carbonyl cyanide *p*-(trifluoromethoxy) phenylhydrazone (FCCP), and thapsigargin. (A) Representative Western blot analysis of phospho-eIF2α (p-eIF2α) abundance (*n* = 3). (B) Quantification of p-eIF2α density intensities. Values were normalized to the loading control β-actin density values and are shown relative to untreated cells (*n* = 3). (C) Stress induction was also controlled by immunofluorescence analysis of SG formation. SGs were visualized by immunostaining of eIF3B (red) and DENV infection by immunostaining of NS1 (green). Representative fields of view are shown. Yellow squares represent the cropped section shown in the merge panel. Scale bars, 50 µm. (D, E, and F) Similar analyses with naive Huh7 cells and Huh7 cells infected with MR766 (MOI of 10) for 30 h and left untreated or treated with different eIF2α kinase-inducing stressors, including arsenite, FCCP, and thapsigargin. (D) Western blot analysis of phospho-eIF2α (p-eIF2α) abundance (*n* = 3). (E) Quantification of p-eIF2α density intensities (*n* = 3). (F) Control of stress induction by immunofluorescence. ZIKV infection was visualized by immunostaining of dsRNA (green) and SGs by immunostaining of eIF3B (red). Download Figure S5, PDF file, 1.3 MB. Copyright © 2017 Roth et al. 2017 Roth et al. This content is distributed under the terms of the Creative Commons Attribution 4.0 International license.

While the above-described experiments excluded a role for PKR in DENV-induced eIF2α phosphorylation, they did not address a potential role of PKR in translation repression. To test this, we verified by siRNA-mediated gene silencing that transient PKR suppression did not affect DENV replication (see Fig. S6A and S6B in the supplemental material) and then established Huh7-derived PKR knockout (ko) cell clones. Three ko cell clones (2#2, 2#3, and 3#1) were selected that lacked basal levels of PKR and failed to produce detectable amounts of PKR upon induction with interferon alpha (62) (Fig. S6C). Polysome profiles of Huh7 PKR ko cell clone 2#3, in which DENV replication levels were similar to those of parental cells (Fig. S6D), showed a similar reduction of polysomal RNAs throughout the course of infection (Fig. 4C), as observed for the parental Huh7 cells (Fig. 1B). Similar results were obtained with Huh7 PKR ko cell clones 2#2 and 3#1. This suggests that PKR activation during DENV infection is not required for translation suppression in human Huh7 cells.

10.1128/mBio.02150-16.7 Figure S6  Impact of PKR on DENV replication. (A and B) Transient PKR silencing does not alter DENV replication. *In vitro* transcript of DENV firefly luciferase reporter virus (50) was coexpressed with nontargeting siRNA (siNT) or PKR-specific siRNA in Huh7 cells. Cells were harvested at 4, 24, 48, and 72 h postelectroporation, and PKR mRNA levels were quantified at 72 h by qRT-PCR (A). All values were normalized to GAPDH mRNA levels. Results represent fold induction relative to cells transfected with siNT. Shown are means of triplicate measurements ± SD from a representative experiment (*n* = 3). (B) To assess DENV RNA replication, cells were lysed at the time points specified and firefly luciferase activities were determined (relative light units [RLU]). Values were normalized to the 4 h (input RNA) value. Shown are mean RLU values ± SD from three independent experiments. (C) Representative Western blot analysis (*n* = 3) of PKR abundance in Huh7 PKR ko cell clones upon stimulation with IFN-α. Three different clones of Huh7 PKR ko cells (2#2, 2#3, and 3#1) were left untreated or treated with 1,000 IU/ml IFN-α for 24 h. Huh7 parental cells served as a control. (D) RNA replication of DENV firefly luciferase reporter virus in Huh7 PKR ko cell clones (2#2, 2#3, and 3#1) was analyzed as described in panel B. Shown are mean RLU values ± SD from three independent experiments. (E) ZIKV MR766-induced translational repression is PKR independent. Polysome profiles of naive Huh7 PKR ko cell clone 2#3 (Mock) and Huh7 PKR ko cell clone 2#3 infected with ZIKV MR766 (MOI of 10) were recorded at the indicated times. Shown are representative polysome profile analyses (lower panel) and mean percentages of polysomal ribosomes ± SEM (upper panel). The number of profiles analyzed (*n*) is given at the top. (F) ZIKV-induced SG-like foci are PKR independent. Huh7 PKR ko cell clone 2#3 was infected with ZIKV (MOI of 0.5) for 24 h. Parental Huh7 cells were used as a control. ZIKV infection was visualized by immunostaining of dsRNA (green) and SGs by immunostaining of eIF3B (red) or TIAR (red). Representative fields of view are shown. Yellow squares represent the cropped section shown in the merge panel. Scale bars, 50 µm. Download Figure S6, PDF file, 0.4 MB. Copyright © 2017 Roth et al. 2017 Roth et al. This content is distributed under the terms of the Creative Commons Attribution 4.0 International license.

Growth arrest and DNA-damage-inducible 34 (GADD34), a regulatory subunit protein of phosphatase PP1, is induced in response to eIF2α phosphorylation. GADD34 promotes dephosphorylation of eIF2α and thereby serves as negative-feedback mechanism to trigger recovery from the translation arrest (63–65). GADD34 mRNA levels were upregulated in the course of late DENV infection (see Fig. S7A in the supplemental material), consistent with our earlier report (56). However, at 18 h p.i., when DENV-induced translational repression starts, levels were only moderately upregulated. We therefore hypothesized that early p.i., GADD34 levels are insufficient to antagonize the virus-induced host translation repression. To address this possibility, we used Huh7 cell pools that stably overexpress GADD34 (56) (Fig. S7B). We tested their ability to antagonize arsenite-induced translation inhibition by polysome profile analysis. While Huh7 control cells responded to increasing arsenite concentrations by a strong translational repression (6.5% of polysomal ribosomes at 500 µM [Fig. S7C]), Huh7 GADD34-expressing cells efficiently antagonized the translation block (42.6% of polysomal ribosomes at 500 µM [Fig. S7D]). In contrast, polysomes were similarly reduced in DENV-infected Huh7 control and GADD34-expressing cells (Fig. 4D). For control, GADD34 overexpression was found to reduce DENV replication only slightly (Fig. S7E). Polysome profiles and immunofluorescence analyses of Huh7 PKR ko cells (Fig. S6E and F), as well as Huh7 GADD34-expressing cells (Fig. S7F and G), confirmed that both SG-like focus formation and translation repression upon ZIKV infection do not require the PKR-eIF2α signaling pathway. Hence, flavivirus suppresses translation through a pathway that is independent of the canonical PKR-eIF2α cascade.

10.1128/mBio.02150-16.8 Figure S7  Impact of GADD34 ectopic expression on DENV infection. (A) DENV infection induces GADD34 mRNA levels. Quantification of GADD34 mRNA levels by qRT-PCR in Huh7 cells infected with DENV for 18, 24, and 36 h. All values were normalized to GAPDH mRNA levels. Results represent fold induction relative to naive cells. Shown are means of triplicate measurements ± SD from a representative experiment. (B, C, D, and E) Characterization of GADD34-overexpressing cells. (B) Quantification of GADD34 mRNA in parental Huh7 (Ctrl) and GADD34 cells normalized to GAPDH mRNA levels. Results represent fold induction relative to Ctrl cells. Shown are means of triplicate measurements ± SD from a representative experiment. (C and D) Ectopic expression of GADD34 antagonizes arsenite-induced translation repression. Huh7 Ctrl (C) and GADD34-expressing (D) cells were treated with increasing concentrations of arsenite (50, 125, 250, and 500 µM) for 45 min. Shown are representative polysome profile analyses (lower panels) and mean percentages of polysomal ribosomes ± SEM (upper panels). The number of profiles analyzed (*n*) is given at the top. (E) Ectopic expression of GADD34 reduces DENV replication. Huh7 Ctrl and GADD34 cells were infected with DENV (MOI of 0.01) and supernatants harvested at 24, 48, 72, and 96 h postinfection. Virus titers were determined by plaque assay (PFU per milliliter). (F) ZIKV MR766-induced host translational repression is eIF2α independent. Polysome profiles of Huh7 control cells (Ctrl) and Huh7 cells expressing stably GADD34 (GADD34) infected with ZIKV MR766 (MOI of 10) were recorded 24 h postinfection. Naive cells (Mock) were used as a control. Shown are representative polysome profile analyses (lower panel) and mean percentages of polysomal ribosomes ± SEM (upper panel). The number of profiles analyzed (*n*) is given at the top. (G) ZIKV-induced SG-like foci are phospho-eIF2α-independent. Huh7 cells overexpressing GADD34 and Huh7 Ctrl cells were infected with ZIKV (MOI of 0.5) for 24 h. ZIKV infection was visualized by immunostaining of dsRNA (green) and SGs by immunostaining of eIF3B (red) or TIAR (red). Representative fields of view are shown. Yellow squares represent the cropped section shown in the merge panel. Scale bars, 50 µm. Download Figure S7, PDF file, 0.6 MB. Copyright © 2017 Roth et al. 2017 Roth et al. This content is distributed under the terms of the Creative Commons Attribution 4.0 International license.

### DENV infection does not impair assembly of the cap-eIF4F complex.

**FIG 5:**
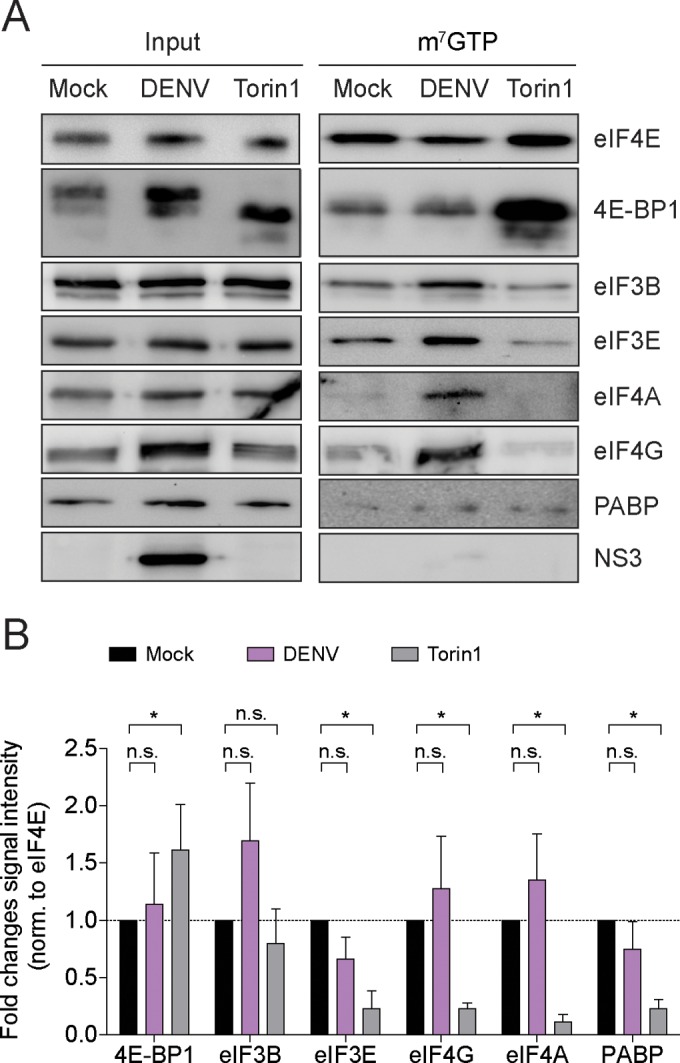
Cap-binding complex assembly is not affected by DENV infection. (A) m^7^GTP immunoprecipitation from naive Huh7 cells or cells infected with DENV (MOI of 10) for 24 h. Cells treated with Torin1 for 16 h were used as control of cap-binding complex disassembly. Shown is representative Western blot analysis of cap-binding proteins coimmunoprecipitated with m^7^GTP-immobilized agarose beads. Shown are input cell extracts (1% of total, left panel) and immunoprecipitated proteins (25% of eluate, right panel). (B) Quantification of cap-binding proteins associated with m^7^GTP. Shown are means ± SEM of fold changes (*n* = 3). *, *P* < 0.05; n.s., not significant.

Since translation suppression in DENV-infected cells did not result from eIF2α inactivation, we next explored whether DENV infection impairs alternative regulators of translation initiation: e.g., the ability to assemble the cap-eIF4F complex (66). To address this question, cap-binding proteins were isolated by immunoprecipitation from naive and DENV-infected Huh7 cells using immobilized m^7^GTP (Fig. 5A). As a control for cap disassembly, Huh7 cells were treated with Torin1, an mTOR inhibitor that blocks 4E-BP1 phosphorylation and thereby lowers the availability of eIF4E for translation (67). As expected, treatment of Huh7 cells with Torin1 resulted in hypophosphorylation and increased association of 4E-BP1 with m^7^GTP, whereas binding of eIF4A, eIF4G, eIF3B, eIF3E, and PABP to the cap was decreased (Fig. 5A and B). However, DENV infection did not alter 4E-BP1 phosphorylation or cause a decrease of initiation factor binding to the cap (Fig. 5A and B). Thus, DENV infection does not suppress translation initiation by the disassembly of the eIF4E-cap-binding complex.

### DENV requires activation of the p38-Mnk1 kinase pathway for virus production.

**FIG 6:**
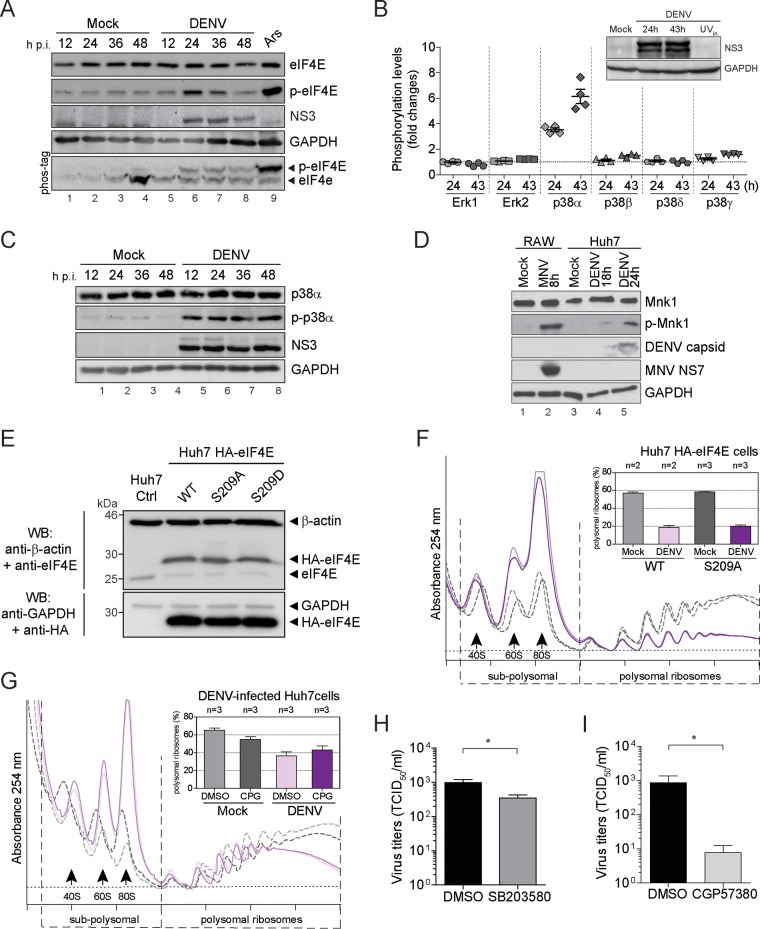
Activation of the p38-Mnk1 signaling pathway is required for virus production. (A) eIF4E phosphorylation levels increase in DENV-infected cells. Shown is representative Western blot analysis (*n* = 3) of phospho-eIF4E (p-eIF4E) abundance in naive and DENV-infected Huh7 cells at 12, 24, 36, and 48 h p.i. Phosphorylation of eIF4E was analyzed by Phos-tag acrylamide gel (lower panel). (B) Analysis of MAPK phosphorylation levels in Huh7 cells inoculated with UV-inactivated DENV (Ctrl) or infected with DENV for 24 and 43 h by using the Proteome Profiler human phospho-MAPK array. Shown in the lower panel are mean relative pixel densities normalized to the control of two independent experiments with two measurements each. (Upper panel) Representative Western blot analysis (*n* = 2) of Huh7 cell extracts used for analysis of MAPK phosphorylations. Naive Huh7 cells, Huh7 cells infected with DENV for 24 and 43 h, and Huh7 cells inoculated with UV-inactivated DENV for 43 h were analyzed. (C) Representative Western blot analysis of phospho-p38α (p-p38α) abundance in naive and DENV-infected Huh7 cells (*n* = 3). (D) Representative Western blot analysis of phospho-Mnk1 (p-Mnk1) abundance in naive and DENV-infected Huh7 cells (*n* = 2). Lysates of naive and murine norovirus (MNV)-infected mouse leukemic monocytes/macrophages (RAW264.7) served as a positive control (72). (E, F, and G) Phosphorylation of eIF4E is dispensable for DENV-induced repression of translation. (E) Ectopic expression of HA-tagged eIF4E in Huh7 cells. Shown is representative Western blot (WB) analysis (*n* = 3) of endogenous eIF4E and HA-eIF4E abundance in Huh7 HA-eIF4E, Huh7 HA-eIF4E(S209A), and Huh7 HA-eIF4E(S209D). Huh7 control (Ctrl) cells served as a control. (Upper panel) Immunoblotting using an anti-eIF4E antibody. (Lower panel) Immunoblotting using an anti-HA antibody. (F and G) DENV-induced translational repression is phospho-eIF4E independent. (F) Polysome profiles of Huh7 cells stably expressing HA-eIF4E (WT) and the mutant HA-eIF4E(S209A) infected with DENV (MOI of 10) were recorded 24 h p.i. Naive cells (Mock) were used as a control. Shown are representative polysome profile analyses (lower panel) and mean percentages of polysomal ribosomes ± SEM (upper panel) *n*, number of profiles analyzed. (G) Naive Huh7 cells (Mock) and cells infected with DENV (MOI of 10) for 8 h (DENV) were treated with CGP57380, an inhibitor of Mnk1 phosphorylation, for 16 h. Treatment with DMSO was used as a control. Shown are representative polysome profile analyses (lower panel) and mean percentages of polysomal ribosomes ± SEM (upper panel) *n*, number of profiles analyzed. (H) Inhibition of p38α reduces DENV virus production. Huh7 cells were infected with DENV (MOI of 0.1) and cotreated with DMSO or 50 µM SB203580, an inhibitor of p38α activity. Virus titers were determined by limiting dilution assay. Shown is the mean ± SD (*n* = 3). *, *P* < 0.05. (I) Inhibition of Mnk1 phosphorylation severely diminishes DENV virus production. Huh7 cells were infected with DENV (MOI of 0.1) for 8 h and subsequently treated with DMSO or 50 µM CGP57380 for 16 h. Virus titers were determined by limiting dilution assay (TCID_50_ per milliliter). Shown is the mean ± SD (*n* = 4). *, *P* < 0.05.

Association of eIF4E with eIF4G within the cap-binding complex is important for cap recognition and for the regulation of eIF4E phosphorylation (68). In response to activation of the MAPKs p38 and extracellular signal-regulated kinase (ERK), the MAP kinase-interacting kinases Mnk1/2, when bound to the eIF4E-eIF4G complex, phosphorylate eIF4E on serine residue 209 (17, 69). Several viruses are known to hijack this pathway or interfere with eIF4E dephosphorylation (10). Thus, we investigated the phosphorylation status of eIF4E during DENV infection. eIF4E phosphorylation showed a transient increase at 24 h p.i. and returned to basal levels at 48 h p.i. (Fig. 6A). Induction of eIF4E phosphorylation upon DENV infection was also observed using Phos-tag acrylamide gel electrophoresis (Fig. 6A, lower panel). eIF4E is phosphorylated by Mnk1/2 downstream of MAPKs p38 and ERK (70, 71). We therefore tested whether DENV infection activated the MAPK signaling pathway using a human phospho-MAPK array and examined ERK1/2 and p38 phosphorylation levels at 24 and 43 h p.i. (Fig. 6B). While inoculation with UV-inactivated virus did not trigger MAPK activation, the p38 main isoform, p38α, was strongly phosphorylated at 24 and 43 h post-DENV infection. In contrast, phosphorylation of the other isoforms p38β, p38δ, and p38γ, as well as of ERK1 and ERK2, remained unchanged (Fig. 6B). p38α phosphorylation in DENV-infected cells was confirmed by Western blotting and observed as early as 12 h p.i. (Fig. 6C). Upon activation by p38, Mnk1 is activated to phosphorylate eIF4E (17). Consistent with the activation of p38α, the phosphorylation of the eIF4E upstream kinase Mnk1 was increased at 24 h p.i. (Fig. 6D). However, this phosphorylation was weaker than that previously shown to be induced during murine norovirus (MNV) infection (72).

To test whether phosphorylation of eIF4E was involved in DENV-induced repression of host cell translation, we established Huh7 cell pools overexpressing wild-type hemagglutinin (HA)-tagged eIF4E, phospho-ablative HA-eIF4E(S209A), and phosphomimetic HA-eIF4E(S209D) mutants (71). The characterization of these cells pools showed that ectopic expression of HA-tagged eIF4E variants resulted in reduced expression levels of endogenous eIF4E compared to control cells (Fig. 6E), reflecting the tight regulation control of eIF4E expression levels (73). Importantly, addition of an HA tag at the N terminus of eIF4E variants did not impair eIF4E association with polysomes (see Fig. S8A in the supplemental material). Finally, ectopic expression of eIF4E variants did not affect rates of translation of Huh7 cell pools compared to those of parental cells (Fig. S8A and B). Stable overexpression of the phosphomimetic HA-eIF4E(S209D) mutant in naive Huh7 cells did not reduce translation rates (Fig. S8B), providing the first evidence that eIF4E phosphorylation itself does not induce host translation repression. Comparison of polysome profiles of DENV-infected Huh7 HA-eIF4E wild-type and Huh7 HA-eIF4E(S209A) cells, in which DENV replication levels were similar to those of control cells (Fig. S8C), revealed that overexpression of the phospho-ablative HA-eIF4E(S209A) mutant failed to rescue the host translation repression (Fig. 6F). These results confirm that eIF4E phosphorylation is dispensable for DENV-induced translation repression. Furthermore, inhibition of Mnk1 function using a specific chemical inhibitor of Mnk1 activity, CGP57380 (74, 75) at a noncytotoxic concentration in Huh7 cells (Fig. S8D), prevented eIF4E phosphorylation in naive and DENV-infected Huh7 cells (Fig. S8E) but failed to rescue DENV-induced translation repression (Fig. 6G). Altogether, these results indicate that eIF4E phosphorylation during DENV infection is dispensable for translation suppression and support the current model in which eIF4E phosphorylation would rather favor the translation of selective mRNAs than impair translation initiation on a more global level (15).

10.1128/mBio.02150-16.9 Figure S8  Activation of the p38-Mnk1 pathway. (A and B) Characterization of Huh7 cells stably overexpressing HA-eIF4E wild type (WT), the phospho-ablative mutant HA-eIF4E(S209A) and the phosphomimetic mutant HA-eIF4E(S209D). (A) Representative polysome profiles of Huh7 cells stably overexpressing the HA-tagged eIF4E wild type and S209A and S209D mutants. After separation by ultracentrifugation, sucrose gradients were eluted from the top using a fractionator, and absorption at 254 nm was continuously recorded (upper panel). Fractions were collected (1 to 14), and proteins were purified and analyzed by Western blotting (lower panel) to confirm the presence of HA-tagged eIF4E in polysomal fractions. (B) Shown are mean percentages of polysomal ribosomes ± SEM (upper panels). The number of profiles analyzed (*n*) is given at the top. (C) Stable overexpression of HA-tagged eIF4E and mutants does not alter DENV virus production. Huh7 control (Ctrl), HA-eIF4E wild-type (WT), HA-eIF4E(S209A), and HA-eIF4E(S209D) cells were infected at an MOI of 0.1 TCID_50_ per cell for 2 h. Twenty-four, 48, 72, and 96 h postinfection, cells were harvested, and infectious titers were determined by limiting dilution assay (TCID_50_ per milliliter). Shown are mean values ± SD from three independent experiments. (D) Cytotoxicity of CGP57380 treatment. Huh7 cells were treated with increasing concentrations of CGP57380 (2.5, 5, 10, 25, 50, 75, and 100 µM) for 16 h. Cell viability was determined by measuring release of ATP using the CellTiter-Glo assay. DMSO was used as a control. Values were normalized to untreated cells and are represented as percentage of cell survival (*n* = 3). (E) Representative Western blot analysis (*n* = 3) of phospho-eIF4E (p-eIF4E) abundance in Huh7 cells treated with the Mnk1 inhibitor CGP57380. Huh7 cells were infected for 8 h and subsequently treated with 50 μM CGP57380 (CGP) for 16 h or with DMSO or left untreated (Untr.). Naive cells were used as control. (F) Cytotoxicity of SB203580 treatment. Huh7 cells were treated with increasing concentrations of SB203580 for 16 h (2.5, 5, 10, 25, 50, 75, and 100 µM). Cell viability was determined as described for panel B (*n* = 3). (G) Representative Western blot analysis (*n* = 2) of phospho-eIF4E (p-eIF4E) abundance in Huh7 cells treated with p38 inhibitor. Huh7 cells were infected and cotreated with 50 μM SB203580 (SB) for 16 h or with DMSO or left untreated (Untr.). Naive cells were used as a control. Download Figure S8, PDF file, 0.2 MB. Copyright © 2017 Roth et al. 2017 Roth et al. This content is distributed under the terms of the Creative Commons Attribution 4.0 International license.

Our results indicated that eIF4E phosphorylation did not influence DENV replication (see Fig. S8C in the supplemental material). To investigate the importance of the p38-Mnk1 pathway during DENV infection, we analyzed DENV particle production in the presence of the p38 inhibitor SB203580 (76) and of the Mnk1 inhibitor, CGP57380. At a concentration of 50 µM, both SB203580 and CGP57380 did not affect Huh7 cell viability (Fig. S8D and F) and reduced eIF4E phosphorylation levels in DENV-infected Huh7 cells (Fig. S8E and G). Inhibition of p38 by SB203580 treatment resulted in a moderate but significant reduction of DENV infectious titers compared to those in the control DMSO-treated cells (Fig. 6H). Consistently, inhibition of Mnk1 by CGP57380 treatment strongly impaired DENV particle production of approximately 100-fold (Fig. 6I). These results suggest that during DENV infection, the activation of the p38-Mnk1 signaling pathway plays a major role for virus production that is independent of eIF4E phosphorylation.

## DISCUSSION

**FIG 7:**
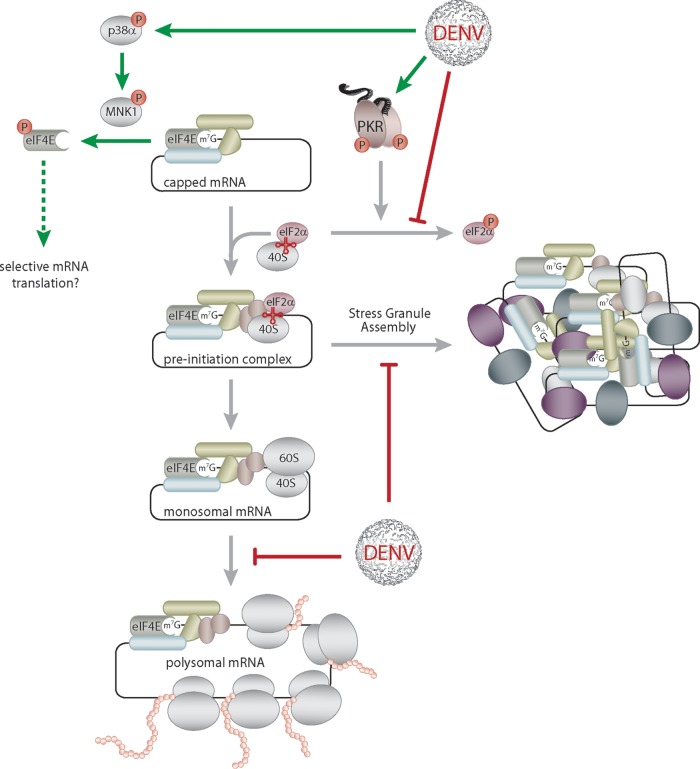
Modifications of host cell translation and stress response by DENV infection. Early in DENV infection, host cell translation is repressed. While assembly of the preinitiation complex and translation elongation remain unaffected, translation initiation is stalled after association of the 60S ribosomal subunit with cellular mRNA (monosomal RNA) (red blunted arrows). Interestingly, DENV infection activates the p38α-Mnk1 pathway, resulting in the phosphorylation of eIF4E (green arrows). While eIF4E phosphorylation does not account for DENV-induced translation repression, it might regulate the selective translation of specific mRNA subsets (green dashed arrow). DENV genome replication occurs through dsRNA intermediates that are sensed by PKR, leading to PKR autophosphorylation and activation (green arrow). However, the downstream phosphorylation of eIF2α, a direct target of the activated PKR, is inhibited as well as the assembly of both eIF2α-dependent and -independent SGs (red blunt-ended arrows). Altogether, during DENV infection translation suppression is uncoupled from the activation of the stress response.

Suppression of host cell translation is a strategy that several viruses, including RNA viruses such as poliovirus, vesicular stomatitis virus, encephalomyocarditis virus, and influenza virus, have developed to favor the translation of their viral genome (reviewed in references 2 and 77). Here, we report an unexpected repression of global cellular protein synthesis by cap-dependent flaviviruses (Fig. 7). Our results support the model of a possible switch in DENV genome translation from cap dependent to cap independent under conditions of translation suppression (40). Importantly, our results indicate that this switch occurs in the context of a natural infection, as reflected by the subsistence of viral RNA association with actively translating ribosomes. Consistently, viral protein levels are not affected by the host translation repression while global protein synthesis is strongly reduced.

Flaviviruses were proposed to prevent rather than to induce host translation shutoff (36, 37, 40). Several technical aspects might explain this difference from our findings, including the use of nonhuman cells, different DENV serotype 2 strains, and time points p.i. chosen for analysis. Of note, polysome profiles of Huh7 cells infected with the DENV serotype 2 Bangkok strain 16681 (40) revealed in our experiments a similar translation repression to that induced by DENV1 infection (see Fig. 1F for reference), which also correlated with reduced cytopathic effect in Huh7 cells (data not shown). Analyses of human cells early p.i. by polysome profiling identified host cell translation repression as a new feature of flavivirus infection.

Flaviviruses actively block eIF2α-mediated stress response at different levels, supposedly to avoid the associated host translation suppression. First, WNV and DENV inhibit SG formation in nonhuman cells (36). Consistently, we observed that DENV and WNV, as well as ZIKV, block SG formation in human Huh7 cells (Fig. 7). Second, infection with DENV was reported to trigger the activation of integrated stress response, phosphorylation of eIF2α through PERK activation, and presumably eIF2α dephosphorylation by the GADD34-PP1 complex (37, 38). However, discrepancies exist about eIF2α phosphorylation and the timing of its regulation. Our results support an absence of eIF2α phosphorylation at late times p.i. as well as the activation of a stress response, as reflected by the upregulation of GADD34 mRNA levels in DENV-infected cells. Importantly, our results demonstrate that activation of eIF2α-dependent stress response is uncoupled from the host translation repression during DENV infection. Strikingly, our results indicate that DENV infection also represses eIF2α-independent SG assembly since SG formation induced with hippuristanol, an inhibitor of eIF4A RNA binding (59), was repressed. Since flaviviruses actively block both eIF2α-dependent and -independent SG formation but override the associated translation suppression, it is tempting to speculate that they thereby avoid the sequestration of their RNA genome into SG or its degradation in SG-associated processing bodies (78, 79).

None of the canonical pathways leading to translation initiation alteration that are usually hijacked by viruses to favor their genome translation (2) was involved in the suppression of host translation upon DENV infection. Neither the eIF2α nor eIF4F-cap-binding complex was altered as translation was repressed by DENV infection. Nevertheless, our study showed that DENV infection activates the p38-Mnk1 signaling pathway, which regulates eIF4E phosphorylation and contributed to DENV particle production. While we ruled out that eIF4E phosphorylation accounted for DENV-induced translation repression, using stable overexpression of the phospho-ablative mutant eIF4E(S209A) or the inhibition of Mnk-1 activity, our results imply that eIF4E phosphorylation may rather play a role in the translational remodeling and control of specific mRNAs encoding proteins associated with cell proliferation, inflammation, and interferon production (14–16), similar to that previously proposed during norovirus infection (72). The p38-Mnk1 signaling pathway activation is known to phosphorylate a number of downstream targets other than eIF4E, including heterogeneous ribonucleoprotein A1 (hnRNPA1) (80). Phosphorylation of hnRNPA1 by Mnk1 results in its disassociation from the tumor necrosis factor alpha (TNF-α) 3′ UTR and promotes the translation of the TNF-α mRNA (81). Interestingly, hnRNPA1 phosphorylation also leads to its recruitment in SG (80) and was recently shown to play an essential role in SG aggregation (82). Whether Mnk1-mediated hnRNPA1 phosphorylation could be involved in the absence of SG formation upon DENV infection remains to be investigated.

While our analysis did not reveal the mechanism by which flaviviruses block host cell translation, several scenarios, not mutually exclusive, might explain this repression. First, DENV genome translation could be favored by an optimized usage of codons with low prevalence in the host cell (83–85), and thereby translation of host mRNAs could be attenuated. Second, DENV infection might limit the availability of the translation machinery components such as ribosomal subunits. Affinity purification and mass spectrometry analyses of DENV-infected cells revealed the interaction of NS1 with over 30 ribosomal proteins such as RPL18 which is required for both viral replication and translation (86). Third, our results indicate that viral replication is dispensable for the induction of translational repression, as illustrated by the absence of puromycin incorporation in single cells expressing DENV polyprotein. Interestingly, expression of the DENV polyprotein in Huh7 cells induces endoplasmic reticulum (ER) membrane rearrangements, as observed in DENV-infected cells (M.C. and R.B., unpublished results). Although we cannot exclude the role of single DENV proteins, it is tempting to speculate that host translational repression might also be a consequence of the ER membrane rearrangements which are essential for virus replication. Altogether, the relevance of the host translation repression for virus production is underscored by the simultaneous targeting of multiple pathways that regulate host translation and by its conservation among all flaviviruses. These findings provide a novel perspective on the role of cap-independent translation as a crucial step of flavivirus life cycle and highlight the importance of unraveling underlying mechanisms. Further analyses of these complex virus-host interactions in human immature dendritic cells, the target cells of DENV at the first site of infection (87), will be required.

## MATERIALS AND METHODS

### Cell culture.

Information about the cell lines is provided in Text S1 in the supplemental material.

10.1128/mBio.02150-16.1 Text S1  Supplemental material and methods. Download Text S1, DOCX file, 0.1 MB. Copyright © 2017 Roth et al. 2017 Roth et al. This content is distributed under the terms of the Creative Commons Attribution 4.0 International license.

### Plasmids.

A description of the plasmids used in this study is provided in Text S1.

### Production of DENV NGC and titration.

Ten micrograms of pDVWSK601 *in vitro* transcript was electroporated in BHK-21 cells. Virus supernatants were collected from day 3 to day 5 postelectroporation. Infectious titers of virus stocks were determined by limiting dilution assay, the protocol of which was adapted from reference 88. Detailed information about *in vitro* transcription protocols, virus production, and titration is provided in Text S1.

### Production of flaviviruses and titration by plaque assay.

DENV serotype 1 (strain Hawaii), DENV serotype 3 (strain H87), and DENV serotype 4 (strain H241) were kindly provided by Progen Biotechnik (Heidelberg, Germany). ZIKV strains MR766 and H/PF/2013 were obtained from the European Virus Archive (EVAg [Marseille, France]). WNV (strain New-York 99) was a kind gift of Jonas Schmidt-Chanasit (Hamberg, Germany). All viruses were passaged once on C6/36 cells, and stocks were prepared by virus amplification in Vero E6 cells. Virus stock titers were determined by plaque assay. Detailed information is provided in Text S1.

### Polysome profile analysis.

Cells were seeded to reach a maximum of 90% confluence on the day of analysis (1 × 10^6^ cells for 24 h of infection). Polysome profile analysis was performed as previously described (89). Prior to lysis, cells were treated with 100 µg/ml cycloheximide (CHX [Sigma-Aldrich]) for 10 min and washed with ice-cold phosphate-buffered saline (PBS) containing 100 µg/ml CHX. Cells were lysed by scraping with 200 µl polysome lysis buffer and cleared by centrifugation at 10,000 rpm for 10 min at 4°C. Lysates were loaded onto a linear gradient of 17.5 to 50% and subjected to ultracentrifugation at 35,000 rpm at 4°C using an SW60 rotor (Beckman) for 2.5 h. Fractions were eluted from the top using a Teledyne ISCO gradient elution system. Polysome profiles were obtained by measuring the absorbance at 254 nm. Detailed information about the calculation of translation rates and polysome fractionation procedure is provided in Text S1.

### Ribopuromycylation assay and quantification of fluorescence intensities.

*De novo*-synthesized proteins were quantified by measuring the incorporation of puromycin on native peptide chains as described previously (46, 47). Detailed information is provided in Text S1.
