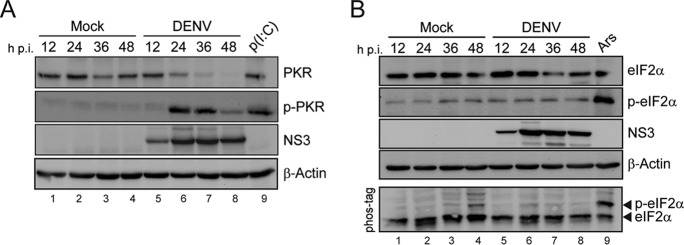# Erratum for Roth et al., “Flavivirus Infection Uncouples Translation Suppression from Cellular Stress Responses”

**DOI:** 10.1128/mBio.00488-17

**Published:** 2017-04-18

**Authors:** Hanna Roth, Vera Magg, Fabian Uch, Pascal Mutz, Philipp Klein, Katharina Haneke, Volker Lohmann, Ralf Bartenschlager, Oliver T. Fackler, Nicolas Locker, Georg Stoecklin, Alessia Ruggieri

**Affiliations:** a Department of Infectious Diseases, Molecular Virology, University of Heidelberg, Heidelberg, Germany; b Division of Biochemistry I, Center for Biomedicine and Medical Technology Mannheim, Medical Faculty Mannheim, Heidelberg University, Mannheim, Germany; c Center for Molecular Biology of Heidelberg University (ZMBH), Heidelberg, Germany; d Division of Virus-Associated Carcinogenesis, German Cancer Research Center (DKFZ), Heidelberg, Germany; e Department of Infectious Diseases, Integrative Virology, University of Heidelberg, Heidelberg, Germany; f Faculty of Health and Medical Sciences, School of Biosciences and Medicine, University of Surrey, Guildford, United Kingdom

## ERRATUM

Volume 8, no. 1, e02150-16, 2017, https://doi.org/10.1128/mBio.02150-16. In the original version of Fig. 4, panel B, one of the loading controls was annotated “GAPDH” while it should read “β-actin.” In panel A, the loading control shown was inadvertently duplicated and has now been replaced by the correct one. The conclusions of the experiment remain unchanged. We apologize for not detecting and correcting this error before publication.

**Figure:**