# Supplementary figures and images for: Flavivirus Infection Uncouples Translation Suppression from Cellular Stress Responses
Source: mBio. 2017 Jan 10;8(1):e02150-16. doi: 10.1128/mBio.02150-16 (PMC5225315; doi:10.1128/mBio.02150-16)

**A**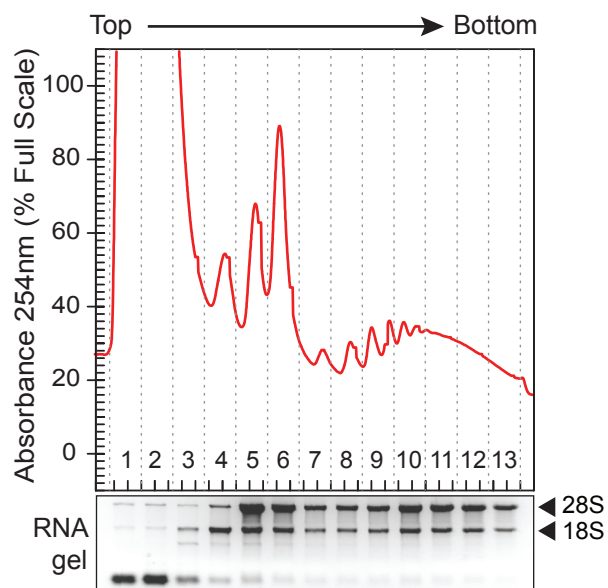**B**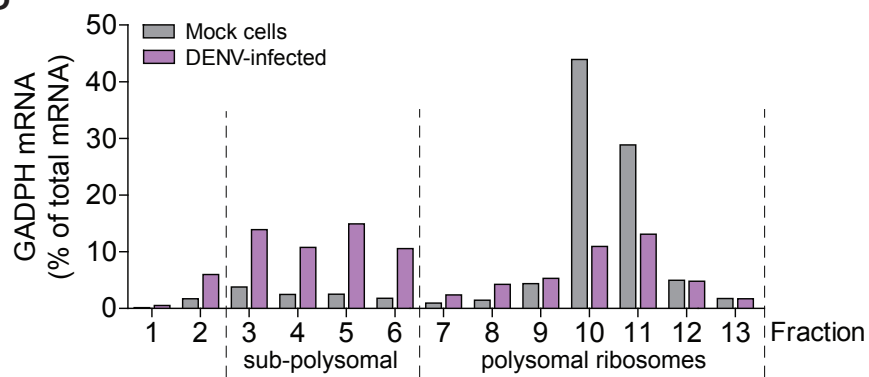**C**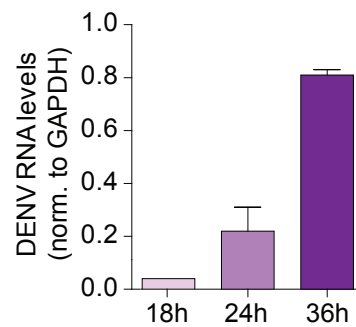**D**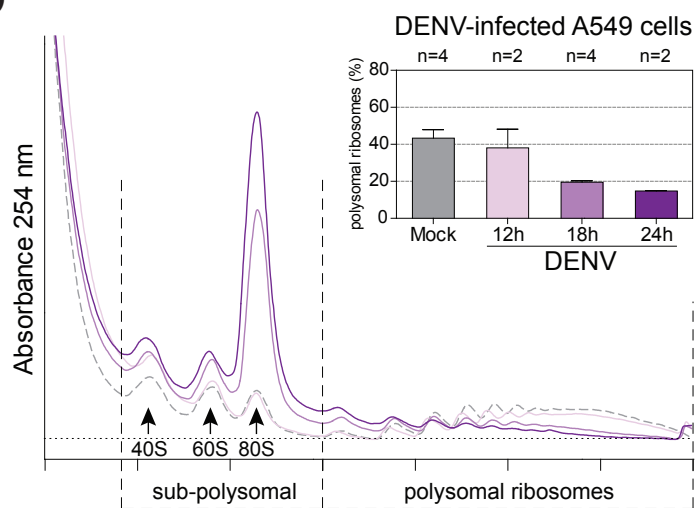**E**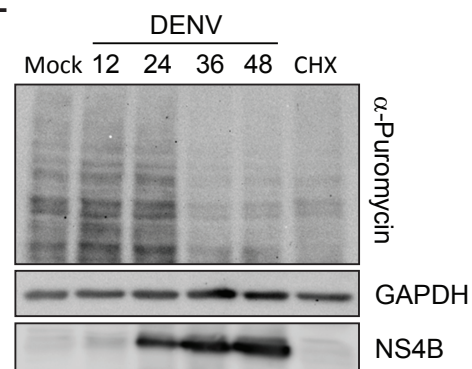

Supplement: Figure S1 [file mbo002173140sf1.pdf]

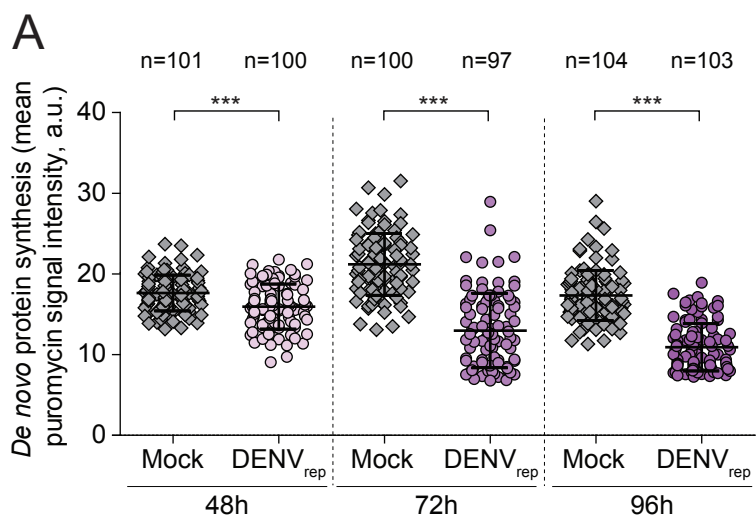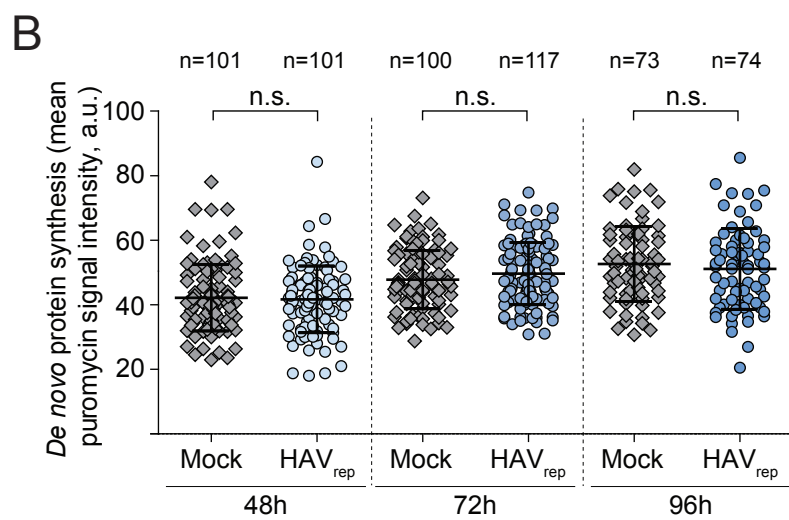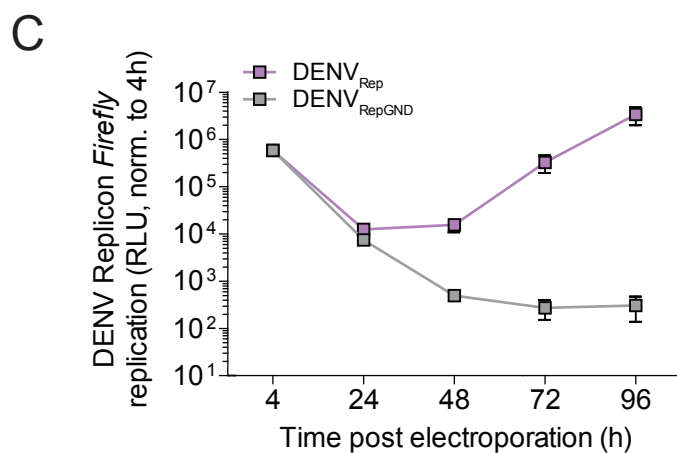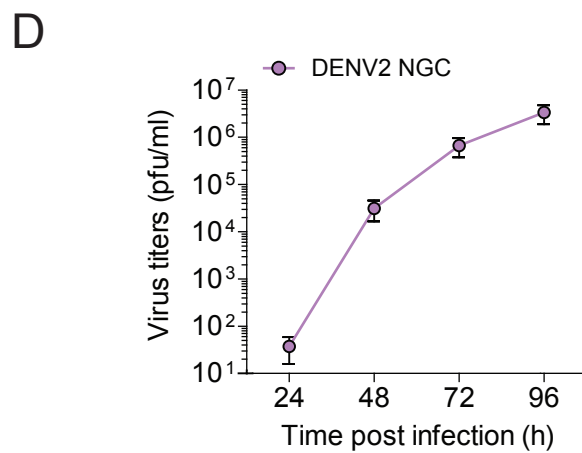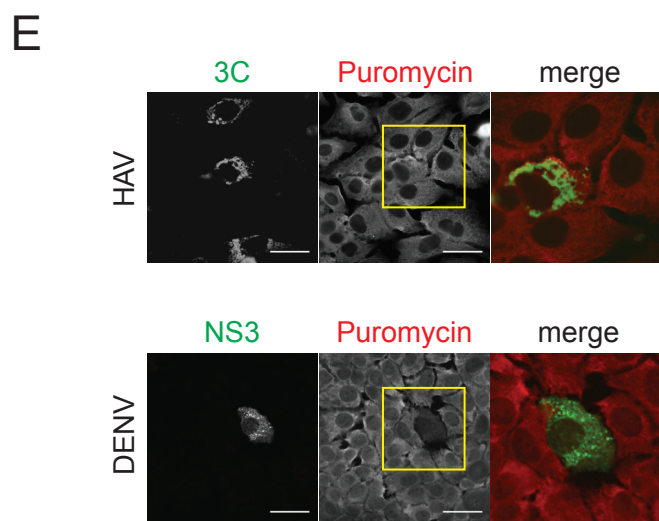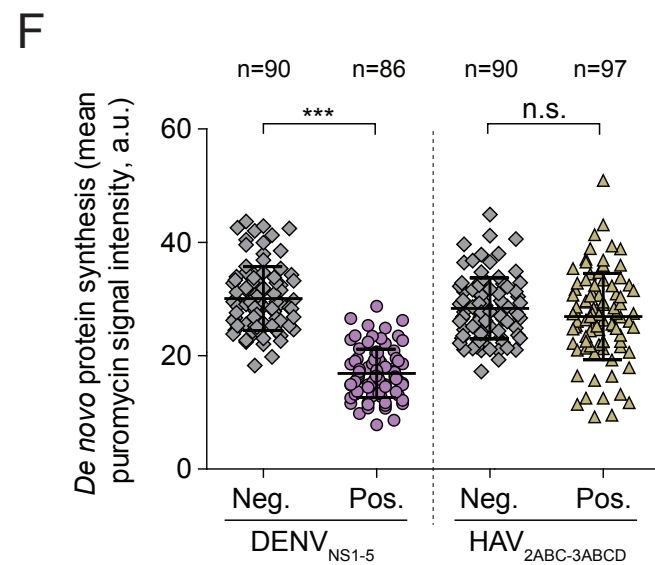

Figure S2

Supplement: Figure S2 [file mbo002173140sf2.pdf]

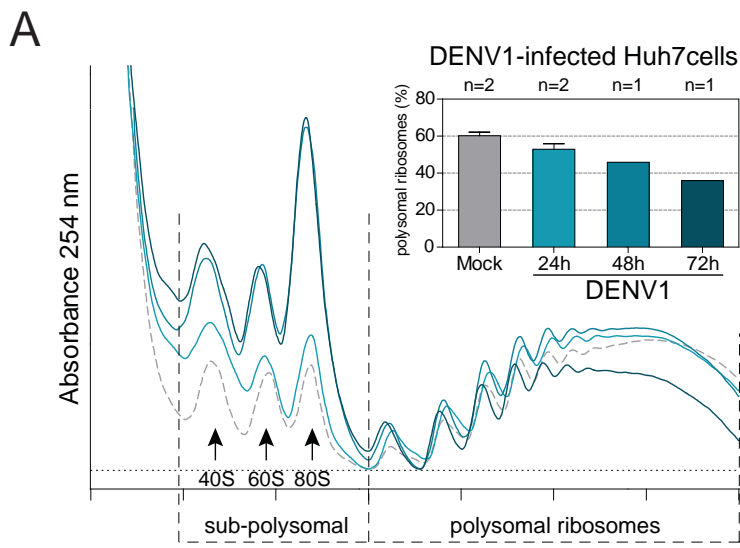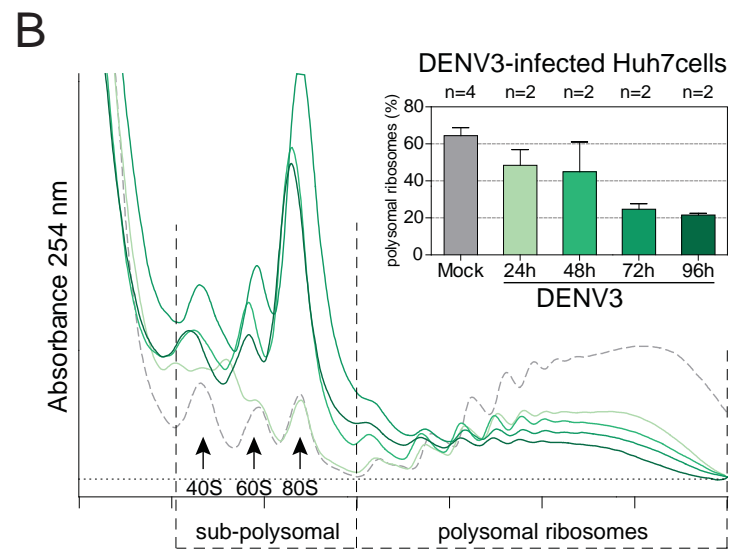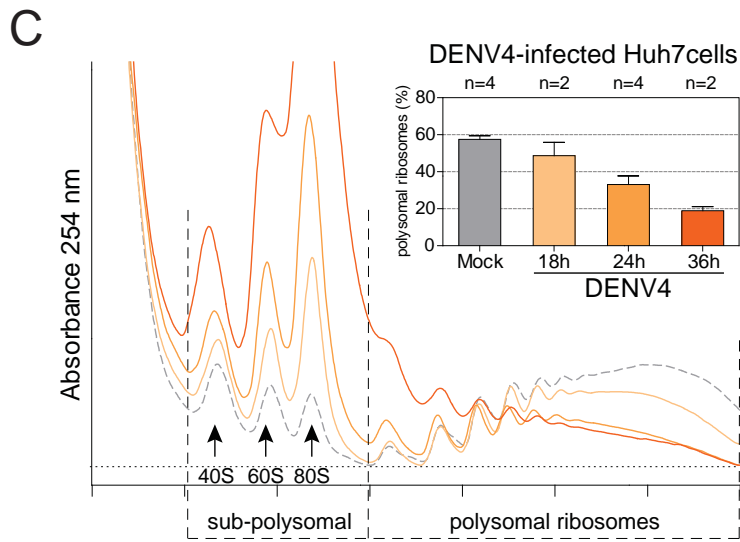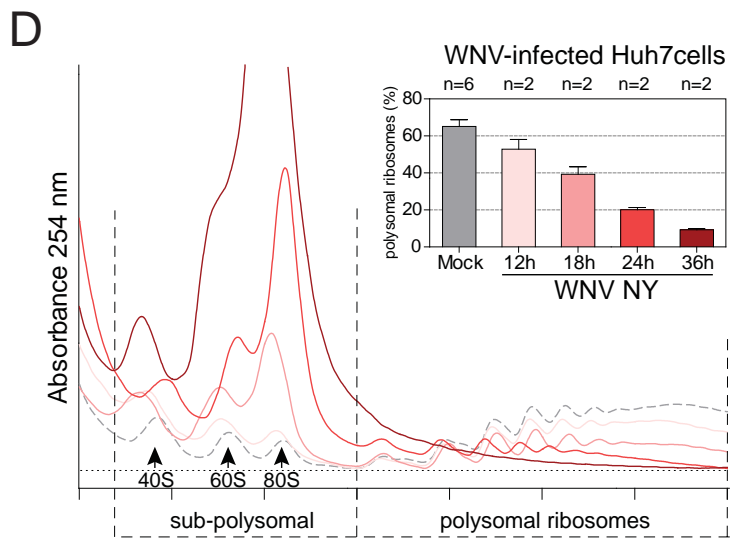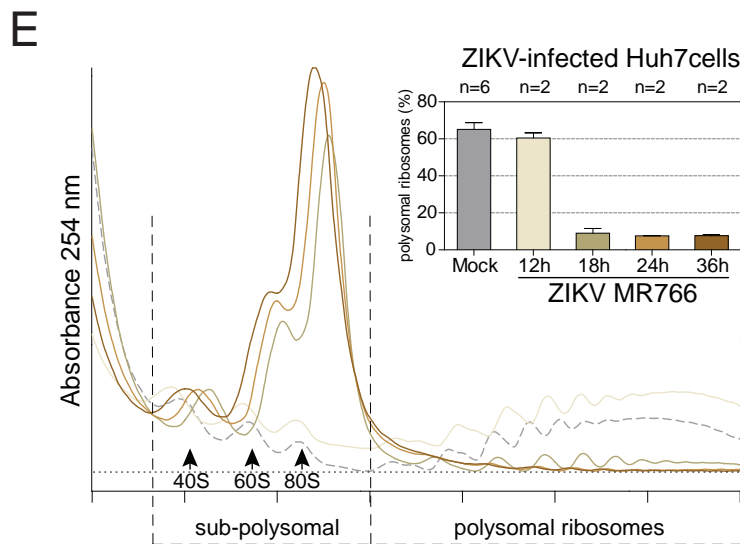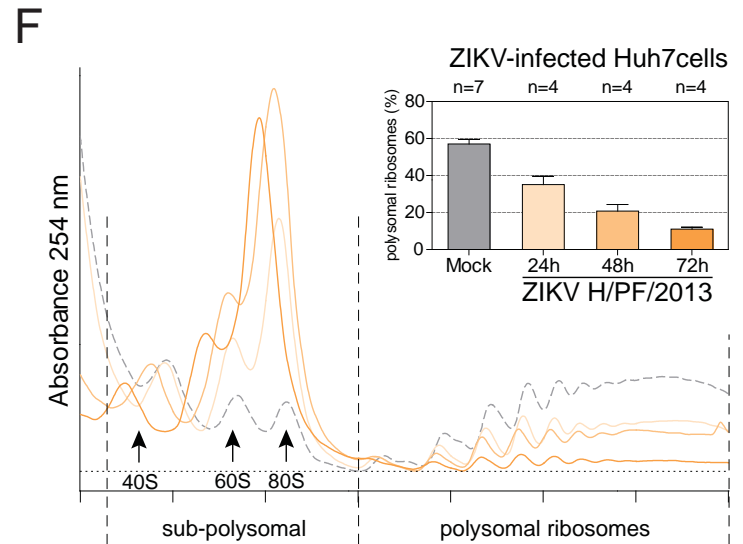

Figure S3

Supplement: Figure S3 [file mbo002173140sf3.pdf]

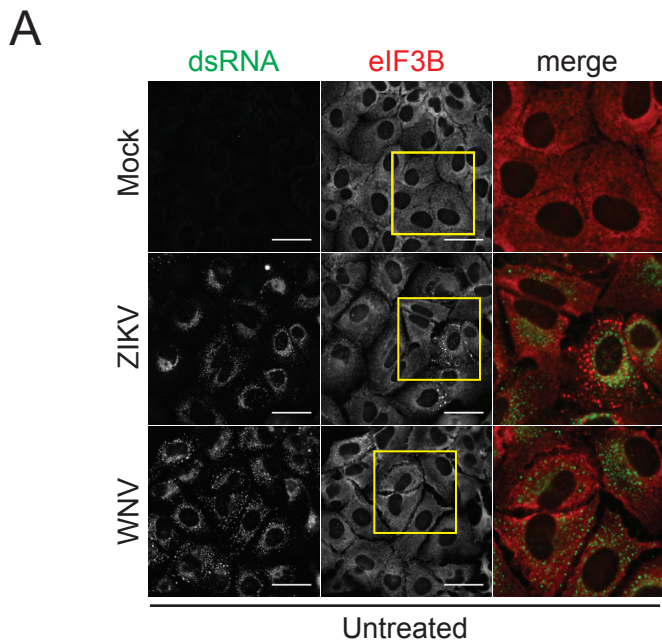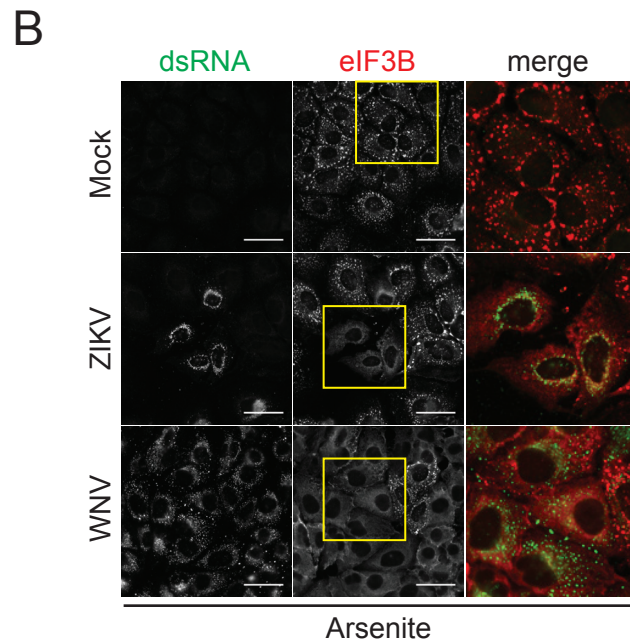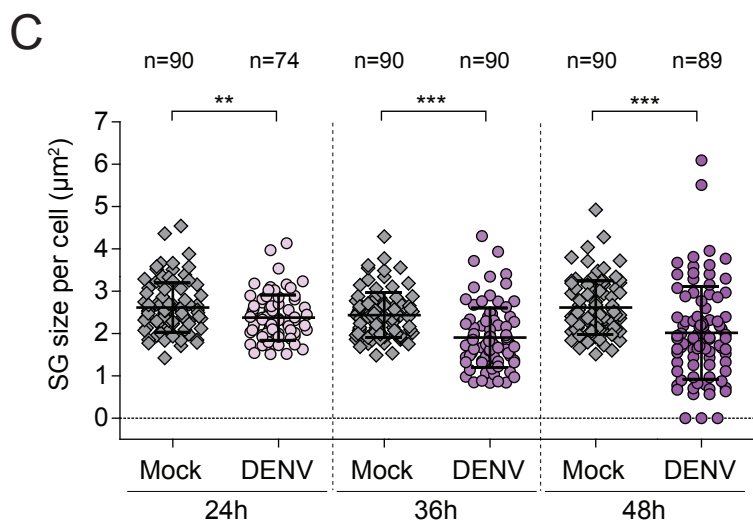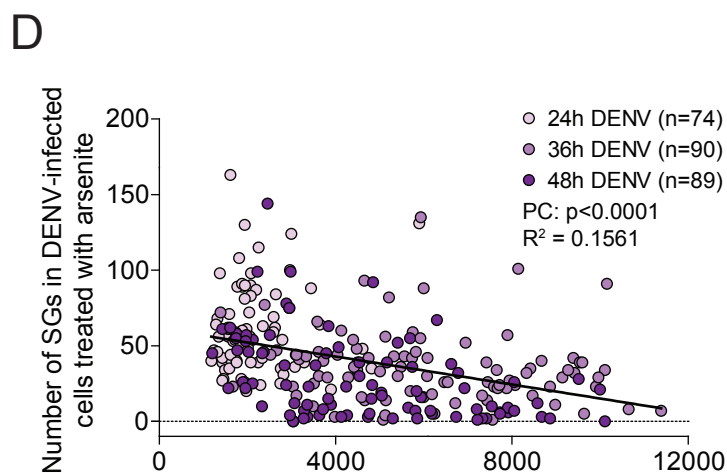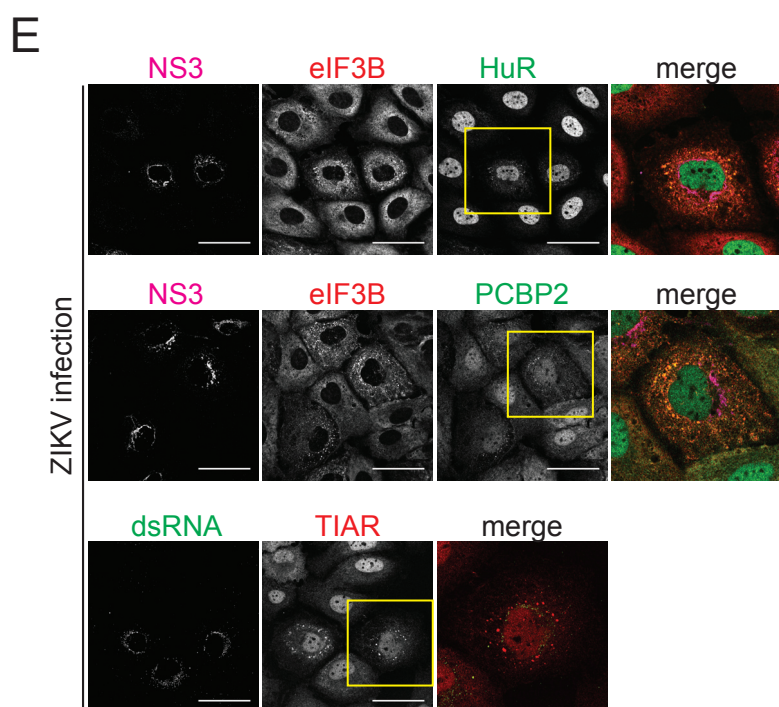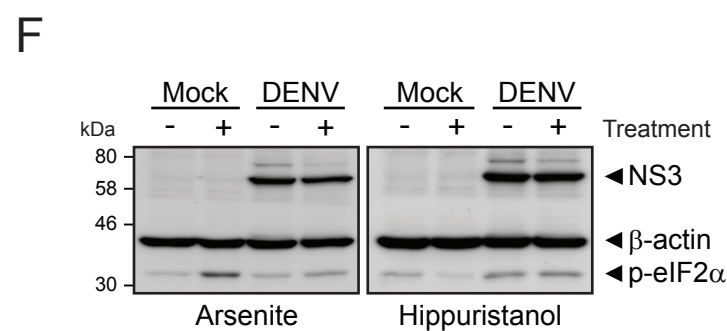

Figure S4

Supplement: Figure S4 [file mbo002173140sf4.pdf]

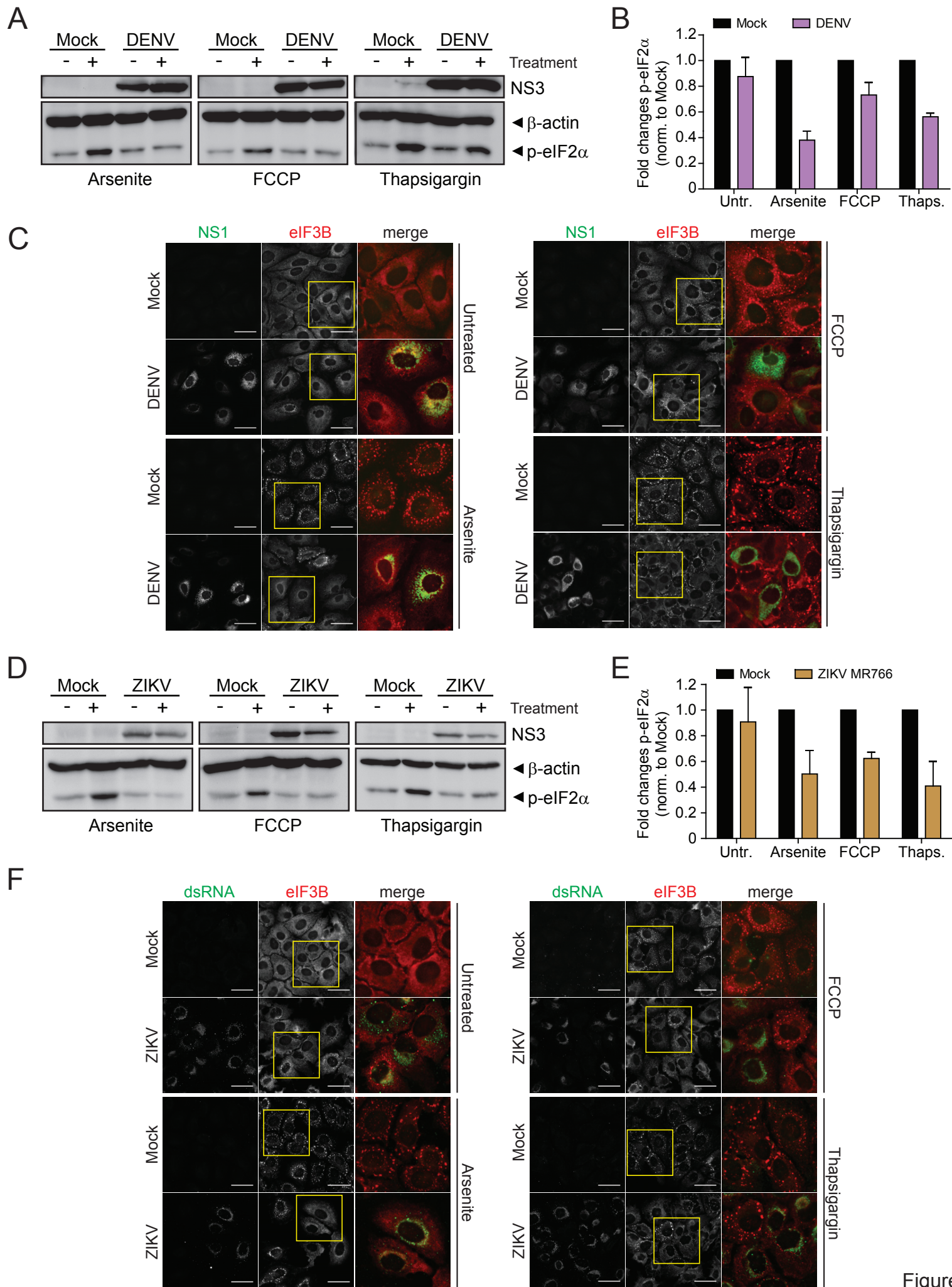

Figure S5

Supplement: Figure S5 [file mbo002173140sf5.pdf]

A

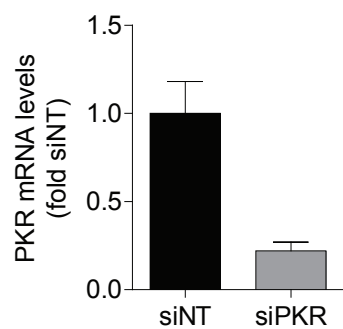

B

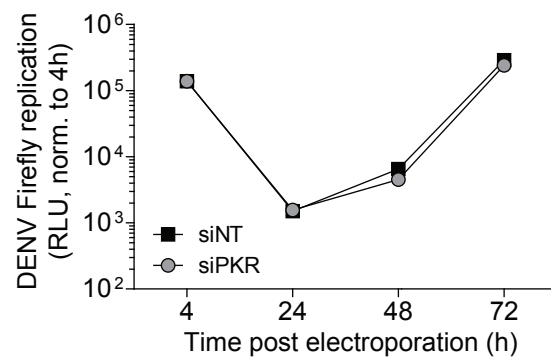

C

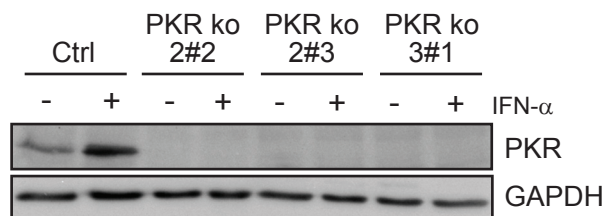

D

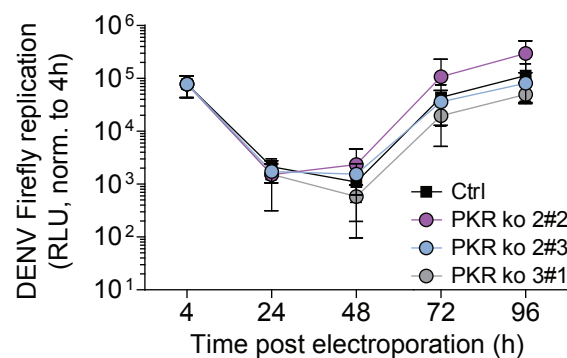

E

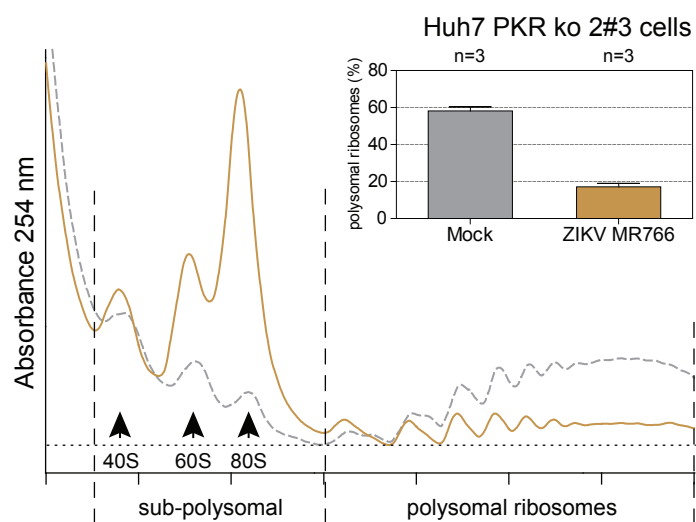

F

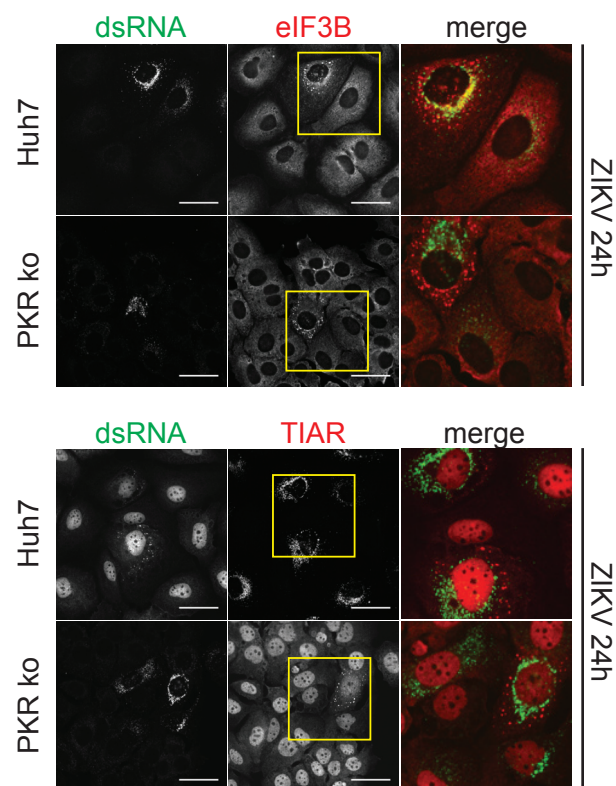

Figure S6

Supplement: Figure S6 [file mbo002173140sf6.pdf]

A

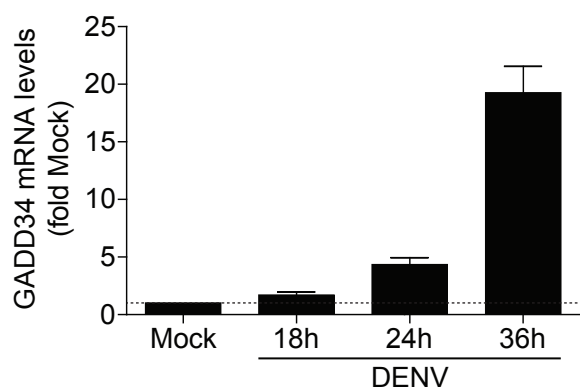

B

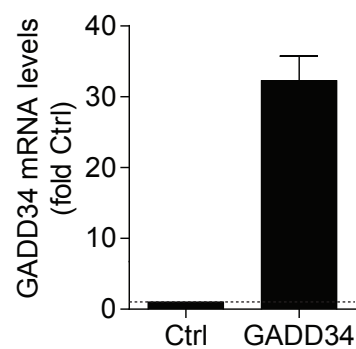

C

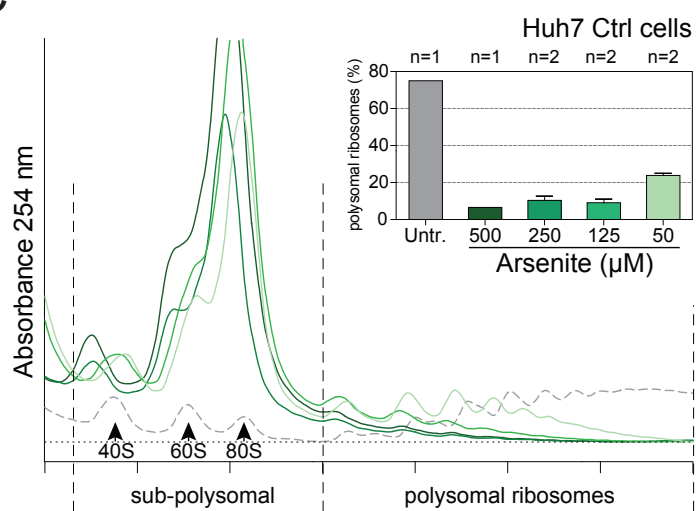

D

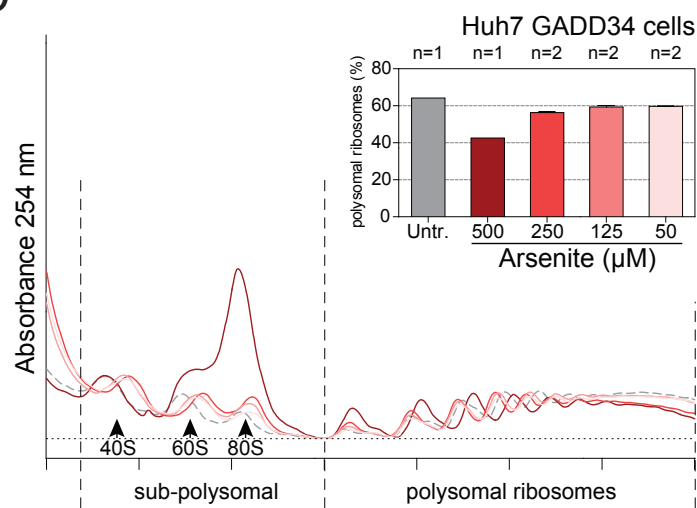

E

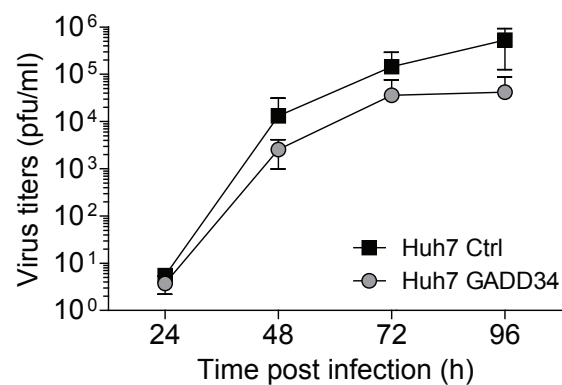

F

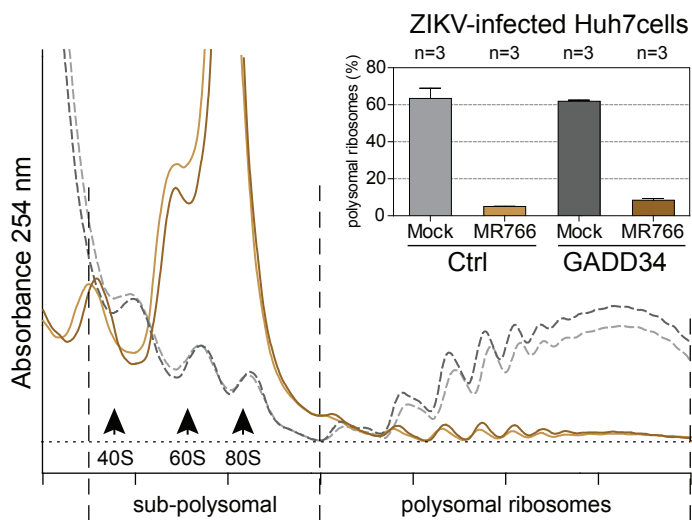

G

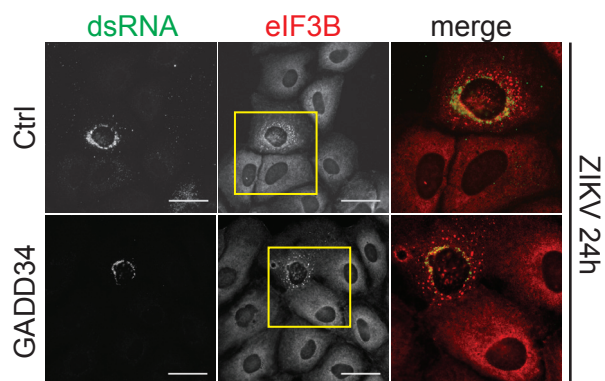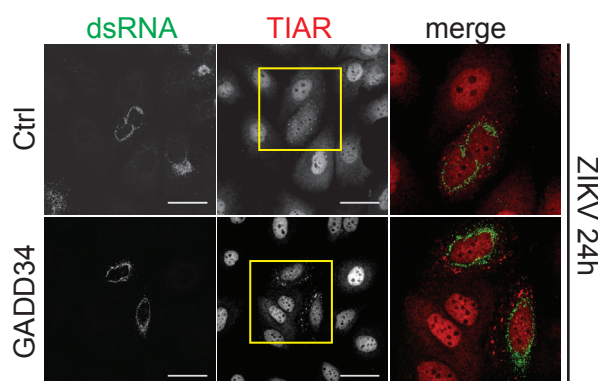

Figure S7

Supplement: Figure S7 [file mbo002173140sf7.pdf]

A

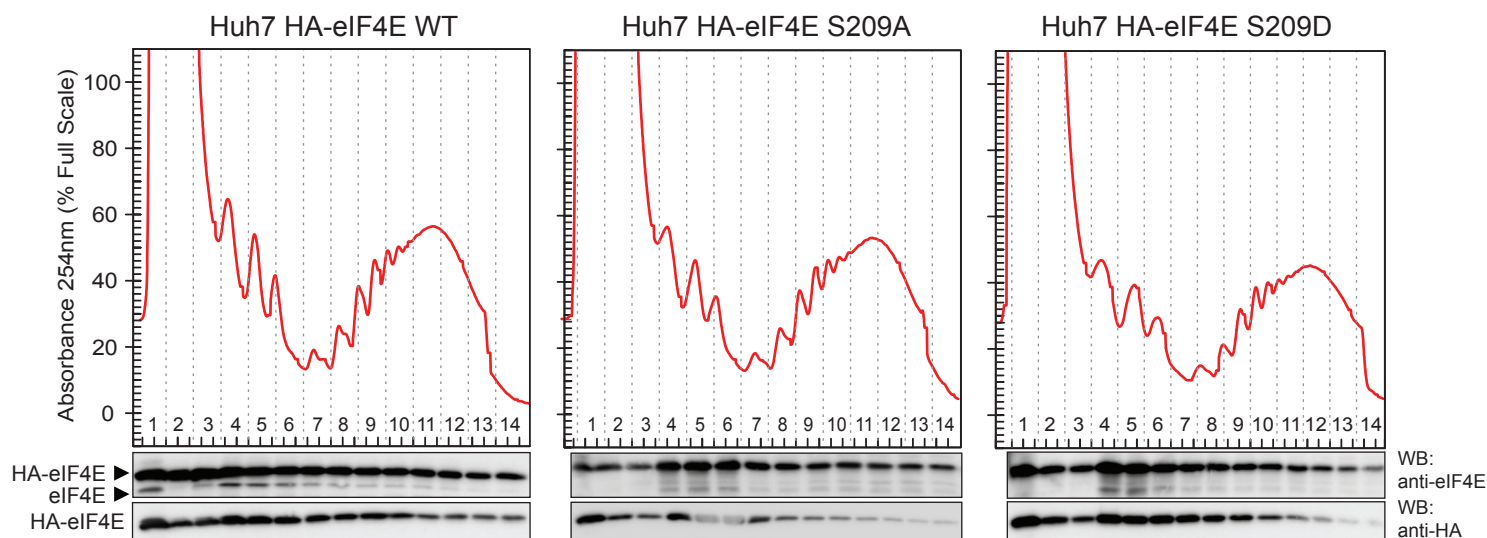

B

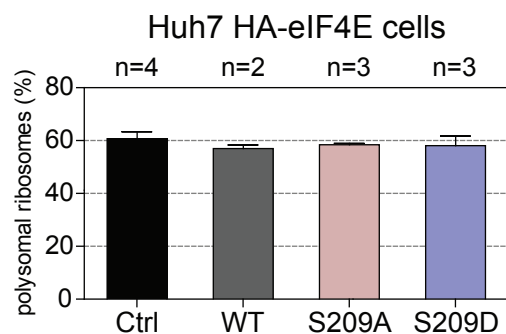

C

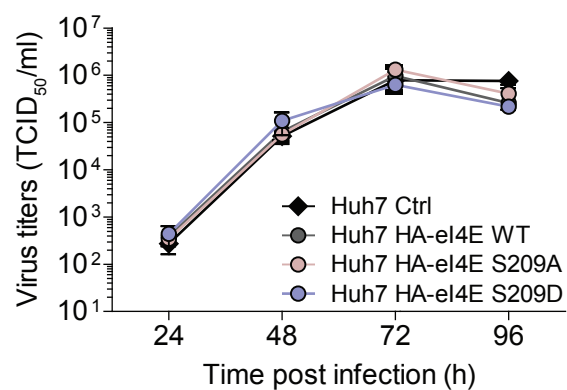

D

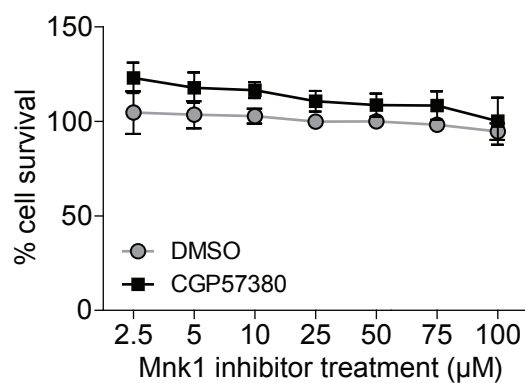

E

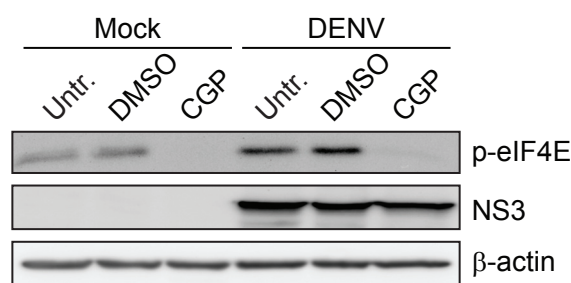

F

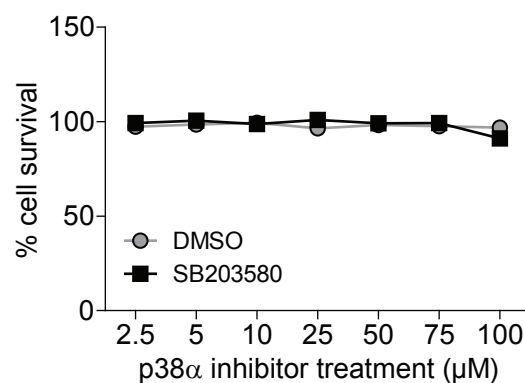

G

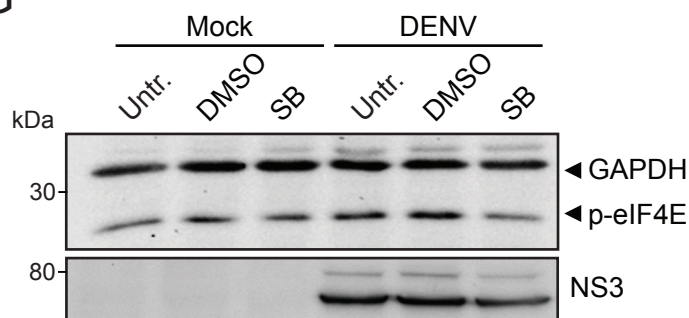

Figure S8

Supplement: Figure S8 [file mbo002173140sf8.pdf]
