# Supplementary material for: Flavivirus Infection Uncouples Translation Suppression from Cellular Stress Responses
Source: mBio. 2017 Jan 10;8(1):e02150-16. doi: 10.1128/mBio.02150-16 (PMC5225315; doi:10.1128/mBio.02150-16)
Supplement: Text S1 [file mbo002173140s1.docx]

***Supplemental Material for***

**Flavivirus infection uncouples translation suppression from cellular stress responses**

Hanna Roth^1^, Vera Magg^1^, Fabian Uch^1^, Pascal Mutz^1^, Philipp Klein^1^, Katharina Haneke^2^, Volker Lohmann^1^, Ralf Bartenschlager^1,3^, Oliver T. Fackler^4^, Nicolas Locker^5^, Georg Stoecklin^2^ and Alessia Ruggieri^1*^.

**Affiliations**

**^1^**Department of Infectious Diseases, Molecular Virology, University of Heidelberg, Heidelberg, Germany

**^2^**Division of Biochemistry I, Center for Biomedicine and Medical Technology Mannheim, Medical Faculty Mannheim, Heidelberg University, Mannheim, Germany; and Center for Molecular Biology of Heidelberg University, Heidelberg, Germany

**^3^**Division of Virus-Associated Carcinogenesis, German Cancer Research Center (DKFZ), Heidelberg, Germany

**^4^**Department of Infectious Diseases, Integrative Virology, University of Heidelberg, Heidelberg, Germany

**^5^**University of Surrey, Faculty of Health and Medical Sciences, School of Biosciences and Medicine, Guildford, United Kingdom

**SUPPLEMENTAL MATERIAL AND METHODS**

**Cell Culture**

Huh7, VeroE6 and A549 cell lines were maintained in Dulbeccos’s modified Eagle´s medium (DMEM) supplemented with 2 mM L-glutamine, 1x non-essential amino acids, 100 U/ml penicillin, 100 µg/ml streptomycin (all from GIBCO, Life Technologies) and 10% fetal calf serum (Sigma-Aldrich). BHK-21 cells were maintained in MEM (Sigma-Aldrich) supplemented with 5% FCS, 10% Tryptose Phosphate Broth (Sigma-Aldrich), 20 mM hepes (Gibco, Life Technologies), 2 mM L-Glutamine, 100 U/ml penicillin, 100 µg/ml streptomycin (all from GIBCO, Life Technologies). Huh7 PKR Blr cells (1), Huh7 HA-eIF4E Blr cells, Huh7 HA-eIF4E S209A Blr, Huh7 HA-eIF4E S209D Blr and Huh7 Blr control cells were additionally supplemented with 5 µg/ml blasticidine (Life Technologies). Huh7 GADD34 Puro cells and Puro control cells (1) were additionally supplemented with 2 µg/ml puromycin (Sigma-Aldrich). Huh7 Lunet T7 Zeo (2) cells were supplemented with 5 µg/ml zeocin (400 µg/ml).

**Plasmids**

The following plasmids were used and are described elsewhere: pDVWSK601, containing the full-length cDNA of DENV type 2 New Guinea C (3, 4) was kindly provided by Andrew Davidson (University of Bristol, England). DENV NGC *Firefly* luciferase reporter virus (pDVWSK601-LucUbi, (5)) was used for characterization of cell line permissiveness and effect of PKR silencing or ko. Wildtype DENV NGC *Firefly* luciferase reporter replicon (pDVWSK601ΔCprME-LucUbi) and replication-defective DENV NGC *Firefly* luciferase reporter replicon (pDVWSK601ΔCprME-LucUbi GND) (5) were used for the ribopuromycylation assay. Hepatitis A virus HM175 18f replicon (6) encoding 2ABC-3ABCD was kindly provided by Yuri Kusov (University of Lübeck, Germany). pTM HAV, encoding for the HAV polyprotein, was generated by transfer of the 2ABC-3ABCD non-structural protein sequence into the expression vector pTM1.2.

**Generation of Huh7 PKR ko Cell Clones by Lentiviral Transduction**

Huh7 PKR knock-out cells were created using the CRISPR/Cas 9 system according to Ran and colleagues (7). Off target and clonal artifacts were avoided by selecting three knock-out clones from two different guide RNAs. In brief, two different guide sequences located on exon 2 of *EIF2AK2* gene were designed using the online open source tool E-CRISP (8). The following sequences were used to design DNA oligos flanked by BsmB1 restriction site overhang sequences: Exon 2.2, 5’- GATGGAAGAGAATTTCCAGA - 3’; Exon 2.3, 5’ - CTCAACAGCTAATTTGGCTG - 3’. DNA oligos were hybridized, phosphorylated and inserted into the lentiviral expression vector lentiCRISPR v2 (Addgene #52961 (9)), encoding for the Cas9 protein.

For production of lentiviral particles 5x10^6^ 293T cells were seeded into 10 cm-diameter dishes and transfected using polyethylenimine (PEI, Polysciences Inc). One hour prior to transfection, medium was replaced. For transfection, 6.4 µg packaging plasmid (pCMVΔ8.91), 6.4 µg transfer vector containing the respective gene of interest and an antibiotic resistance gene (pWPI-based) and 2.1 µg of the VSV envelope glycoprotein expression vector (pMD2.G) were mixed and diluted to a final volume of 400 µl in OptiMEM (GIBCO, Life Technologies). 45 µl PEI was diluted to a final volume of 400 µl in OptiMEM and added to the DNA mix. The mixture was immediately added to the cell culture dish in a drop-wise fashion and the plate was gently swirled to evenly distribute the transfection mixture throughout the plate. After 8h, medium was replaced by 6 ml fresh medium. For stable cells, 6.4x10^5^ target cells were seeded into a 10 cm-diameter dish on the next day. Twenty-four hours later (48h post transfection), supernatant containing lentiviral particles was harvested and replaced by another 4 ml fresh medium. Supernatant was filtered through a 0.45 µm-pore membrane prior to usage. Transduction of target cells with the lentiviral particles was repeated in three times every 12h to achieve high number of integrates and thus high expression levels. Transduced cell pools were subjected to selection with medium containing 1µg/ml puromycin. From the two pools single cell clones were selected by single cell dilution. After colony growth, knock-out efficiency was analyzed by Western blot against PKR expression after stimulation with 1000 IU/ml IFN-α (PBL Laboratories) for 24 h. Cell clones 2#2 and 2#3 were selected from Huh7 cells transduced with lentiviruses expressing the guide RNA 2.2. Cell clone 3#1 was selected from Huh7 cells transduced with lentiviruses expressing the guide RNA 2.3.

**Generation of Huh7 HA-eIF4E Cells by Lentiviral Transduction**

*EIF4E* gene was amplified from the ORFeome cDNA clone library (Invitrogen, Life Technologies) by PCR using the following primers, bearing unique BamHI and XbaI restriction sites, respectively: forward, 5’ - AGTCGGATCCATGGCGACTGTCGAA – 3’, and reverse, 5’ - GACTTCTAGACTAAACAACAAACCT - 3’. The amplified PCR product was treated with the restriction enzymes BamHI and XbaI and inserted in pcDNA3.1 (+) vector treated with the same restriction enzymes. Mutations of the serine residue at position 209 to an alanine (S209A) and an aspartic acid residue (S209D) were introduced using modified reverse primers: S209A reverse, 5’ – GACTTCTAGACTAAACAACAAACCTATTTTTAGTGGTCGCGCCGCT - 3’; S209D reverse, 5’ - GACTTCTAGACTAAACAACAAACCTATTTTTAGTGGTATCGCCGCT - 3’ (mutated sequences are indicated by underlining).

pWPI HA-eIF4E Blr, pWPI HA-eIF4E S209A Blr and pWPI HA-eIF4E S209D Blr generated by amplification of the *EIF4E* gene from the respective pcDNA3.1(+) plasmid using a forward primer bearing the unique AscI restriction site and the HA tag sequence (underlined): 5’ - CTGCAGGCGCGCCATGTACCCATACGACGTCCCAGACTACGCTGGAAGCTTGGTACCGATGGCGACTGTCGAACCGGA - 3’ and reverse primer bearing the unique SpeI restriction site, 5’ - AGTTTACTAGTCTAAACAACAAACCTATTT - 3’. The amplified PCR products were treated with the restriction enzymes AspI and SpeI and inserted into the lentiviral transduction vector pWPI carrying a blasticidin resistance gene treated with the same restriction enzymes.

Production of lentiviral particles for the transduction of Huh7 cells was performed as described above. Transduced cell pools were subjected to selection with medium containing 5µg/ml blasticidin.

***In Vitro* Transcription**

Ten μg of the respective pDVWSK601-based constructs or of HAV 18f replicon construct were linearized by restriction with XbaI or SphI, respectively, and purified using the Nucleospin Extract II kit (Macherey-Nagel). *In vitro* transcription reaction mixtures contained 80 mM hepes (pH 7.5), 12 mM MgCl_2_, 2 mM spermidine, 40 mM dithiothreitol (DTT), 3.125 mM of each nucleoside triphosphate, 1 U of RNasin (Promega), 0.1 μg plasmid DNA, and 0.6 U of T7 RNA polymerase (Promega) per µl reaction. After incubation for 2 h at 37°C, 0.3 U of T7 RNA polymerase per μl reaction mixture was added prior to overnight incubation at 37°C. Transcription was terminated by addition of 1.2 U of RNase-free DNase (Promega) per μg plasmid DNA and 30 min incubation at 37°C. RNA was extracted with acidic phenol and chloroform, precipitated with isopropanol, and dissolved in RNase-free water. RNA integrity was determined by using denaturing agarose gel electrophoresis and concentration was determined by measuring optical density at 260 nm.

***In Vitro* Transcription of eGFP Spike-in and Standard Curve for qRT-PCR Analysis**

Ten µg pTM1.2 eGFP (10) construct were linearized by restriction with XbaI and *in vitro* transcription was performed as described above. 10^3^ to 10^9^ molecules of purified eGFP *in vitro* transcript were used to generate a standard curve for quantification of eGPF transcripts by qRT-PCR after polysome fractionation.

**Cell Electroporation**

Single-cell suspensions of BHK-21 cells were prepared by trypsinization and washed once with phosphate-buffered saline (PBS). Cells were resuspended at a concentration of 1.5×10^7^ cells per ml in Cytomix (11) containing 2 mM ATP and 5 mM glutathione. Ten μg of *in vitro* transcribed RNA was mixed with 400 μl of the cell suspension and transfected by electroporation with a Gene Pulser system (Bio-Rad) in a cuvette with a gap width of 0.4 cm (Bio-Rad) at 975 μF and 270 V. Cells were seeded according to the type of experiment.

**Production of DENV NGC and Titration**

Ten µg of pDVWSK601 *in vitro* transcript were electroporated in BHK-21 cells as described above and immediately transferred into medium and seeded into a 10 cm-diameter culture dish. Virus supernatants were collected from day 3 to day 5 post electroporation, supplemented with 15 mM hepes and filtered through a 0.45 µm-pore membrane. Infectious titers of virus stocks were determined by limiting dilution assay which protocol was adapted from (12). Viral infection was detected by using an immuno-purified DENV anti-E antibody (Hybridoma cell line 3H5-1, ATCC). DENV NGC virus amplification was obtained by infecting 8x10^6^ VeroE6 cells in 15cm-dishes with an MOI of 0.01. Virus supernatants were collected from day 5 to day 8 post infection, supplemented with 15 mM hepes and concentrated by ultrafiltration using a centrifugal filter device (Centricon Plus-70, Millipore) to achieve an around 50-fold volume reduction. Infectious titers of virus stocks were determined by limiting dilution assay using Huh7 cells as mentioned above.

**Production of Flaviviruses and Titration by Plaque Assay**

DENV serotype 1 (strain Hawaii), DENV serotype 3 (strain H87), DENV serotype 4 (strain H241) were kindly provided by Progen Biotechnik (Heidelberg, Germany). ZIKV strains MR766 and H/PF/2013 were obtained from the European Virus Archive (EVAg, France). WNV (strain New-York99) was a kind gift of Jonas Schmidt-Chanasit (Hamburg, Germany). All viruses were passaged once on C6/36 cells and stocks were prepared by virus amplification in VeroE6 cells. Virus stock titers were determined by plaque assay. In brief, VeroE6 cells were infected with serial dilutions of virus supernatants. Two hours post-infection inoculum was replaced by serum-free MEM medium (Gibco, Life Technologies) containing 1.5% carboxymethyl cellulose (Sigma-Aldrich). At different days post infection (day 3 for WNV, day 4 for ZIKV, day 5 for DENV1-3-4 and day 7 for DENV NGC, cells were fixed by addition of formaldehyde to a final concentration of 5%. Cells were stained with crystal violet solution (1% crystal violet, 10% ethanol in H_2_O) for 30 min at room temperature and rinsed extensively with H_2_O. Infectious titers were calculated considering the corresponding dilution factor.

**Replication Analysis and Luciferase Assay**

Quantification of *Firefly* luciferase reporter activity was used to determine transient DENV RNA replication as previously described (5). In brief, 4x10^6^ Huh7 cells were co-electroporated with 5 µg *in vitro* transcript of the DENV *Firefly* luciferase replicon WT and GND, and resuspended in 20 ml medium. To measure the effect of PKR silencing on DENV replication, 5 µg *in vitro* transcript of the DENV *Firefly* luciferase reporter virus (pDVWSK601-LucUbi) and 100 pmol non-targeting siRNA or PKR siRNA (Qiagen *EIF2AK2* #5) were co-electroporated. Two milliliters of the cell suspension were seeded into each well of a 6-well plate in duplicate, and lysed 4, 24, 48, 72 and 96h after electroporation by addition of lysis buffer (0.1% Triton X-100, 25 mM glycyl-glycine, 15 mM MgSO_4_, 4 mM EGTA, and 1 mM DTT, pH 7.8). For detection of *Firefly* luciferase activity, 100 μl lysate were mixed with 360 μl assay buffer (25 mM glycyl-glycine, 15 mM MgSO_4_, 4 mM EGTA, and 15 mM potassium phosphate pH 7.8) containing 1 mM DTT, 2 mM ATP and 70 µM D-luciferin (P.J.K). All measurements were done in duplicate by using a tube luminometer (Berthold). Replication efficiency was determined by normalization to the 4h values reflecting transfection efficiency.

**Infectivity Assays**

To test the permissiveness of different cell lines, Huh7 cells (1x10^5^) were infected at an MOI of 0.01 TCID_50_ per cell for 2 h. Twenty-four, 48, 72 and 96 h post infection, cells were harvested and infectious titers were determined by plaque assay or by limiting dilution assay as described above.

**Polysome Profile Analysis**

Cells were seeded to reach a maximum of 90 percent confluence on the day of analysis (1x10^6^ cells for 24 h infection). Polysome profile analysis was performed as previously described (13). Prior to lysis, cells were treated with 100 µg/ml cycloheximide (CHX, Sigma-Aldrich) for 10 min and washed with ice-cold PBS containing 100 µg/ml CHX. Cells were lysed by scraping with 200 µl polysome lysis buffer (15 mM Tris-HCl pH 7.4, 15 mM MgCl_2_, 300 mM NaCl, 1% Triton X-100, 0,1% β-mercaptoethanol, 100 µg/ml CHX, 0.2 U/ml RNasin (Promega), and EDTA-free protease inhibitor (Roche)). Lysates were tumbled for 10 min at 4°C and cleared by centrifugation at 10,000 rpm for 10 min at 4°C. Ten percent of the cell lysates were kept for input. Lysates were loaded onto a linear gradient of 17.5 to 50% in 15 mM Tris-HCl pH 7.4, 15 mM MgCl_2_, 300 mM NaCl and subjected to ultracentrifugation at 35,000 rpm at 4°C using a SW60 rotor (Beckman) for 2.5 h. Fractions were eluted from the top using a Teledyne ISCO gradient elution system. Polysome profiles were obtained by measuring the absorbance at 254 nm. To quantify the rate of active translation, the recorded curves were aligned according to the lowest values between the 80S peak and the two-ribosome peak. The area under the curve of polysomal ribosomes was calculated and divided by the area of total ribosomes to obtain the percentage of polysomal ribosomes.

**Polysome Fractionation**

Polysome gradients were fractionated over time from the top as mentioned above.

For RNA extraction, fractions were collected every 15 s (approx. 400 µl) in 1.5 ml tubes containing urea buffer and 1 fmol eGFP *in vitro* transcript as spike-in to normalize the samples for RNA extraction loss. Fractions (and input RNA) were denatured in phenol-chlorform-isoamyl alcohol 10 min at 65°C, centrifuged at 13,000 rpm at room temperature for 20 min. The RNA-containing aqueous phase was precipitated in 0.7 volume isopropanol with 1.5 µg/ml of the nucleic acid coprecipitant GlycoBlue (Ambion). RNA pellet was resuspended in 40 µl RNase-free water and stored at -80°C. Five µl of each fraction was loaded on a formaldehyde gel to distinguish fractions with free RNA, monosomal (sub-polysomal) fractions containing 18S ribosomal RNA and polysomal fractions containing 18S and 28S ribosomal RNAs. RNA of each fraction was further processed by qRT-PCR. A serial dilution of purified eGFP *in vitro* transcript was used as standard as described above, and processed in parallel to determine the copy numbers of eGFP spike-in in RNA extracted from polysome fractions. Values were used to correct for RNA loss during RNA extraction.

For protein analysis, fractions were collected every 15 s (approx. 400 µl) in 1.5 ml tubes. Ten µl StrataClean resin (Stratagene, Agilent Technologies, ref: 400715) were added to the collected samples and tumbled overnight at 4°C. Samples were pelleted at 13,000 rpm at 4°C for 5 min. Proteins were eluted in 100 µl 1x Laemmli buffer (62.5 mM Tris-HCl pH 6.8, 10% glycerol, 1.5% SDS, 1.5% β-Mercaptoethanol*,* 0.01% bromophenol blue) by incubating the resin for 5 min at 95°C. Twenty-five µl of the eluate were loaded on a SDS-PAGE for Western blot analysis.

**Quantification of Gene Expression and DENV RNA by qRT-PCR**

Total RNA was extracted by using NucleoSpin RNA II (Macherey-Nagel) according to the manufacturer’s instructions. cDNA was generated using the High Capacity cDNA Reverse Transcription kit (Applied Biosystems). mRNA levels were determined by using the iTaq Universal SYBR Green 2x (Bio-Rad). Reactions were performed on an CFX96 (Bio-Rad) using the following program: 95°C for 3 min and 45 cycles as follows: 95°C for 10 s, 60°C for 30 s.

GAPDH mRNA was used for normalization of input RNA. RT-PCR data were analyzed by using the ΔΔC_T_ method as previously described (14). The following primers were used: GADD34-For 5’ - CAGAAACCCCTACTCATGATCC - 3´, GADD34-Rev 5’ - AAATGGACAGTGACCTTCTCG – 3’; PKR-For 5’ - GCCGCTAAACTTGCATATCTTCA – 3’, PKR-Rev 5’ - TCACACGTAGTAGCAAAAGAACC - 3’; GAPDH-For 5’ - GAAGGTGAAGGTCGGAGTC - 3’, GAPDH-Rev 5’ - GAAGATGGTGATGGGATTTC - 3’; eGFP-For 5’ - TCGTGACCACCCTGACCTAC - 3’, eGFP-Rev 5’ - TGTAGTTGCCGTCGTCCTTG - 3’.

Viral RNA was quantified by qRT-PCR using the qScript XLT One-Step RT-qPCR kit (Quanta Biosciences). In brief, 15 µl of reaction mixture contained 0.6 µl enzyme mixture, 1.5 mM of MgCl_2_, 1.3 µM of each DENV-specific primer (For 5´- GGA AAG ACC AGA GAT CCT GCT GT-3´; Rev 5’ - CATTCCATTTTCTGGCGTTC - 3’), 0.67 mM of each dNTP, 0.27 µM of DENV-specific probe (5’ - 6-FAM - CAGCATCATTCCAGGCACAG - BHQ1 - 3’), 5 µl of template RNA and RNase-free water. To determine absolute RNA amounts, a serial dilution of an RNA standard (10^2^ to 10^8^ HCV RNA copies per reaction) was processed in parallel. Reactions were performed on an CFX96 (Bio-Rad) using the following program: 50°C for 10 min, 95°C for 1 min and 45 cycles as follows: 95°C for 10 s, 60°C for 1 min.

**Ribosome Run-Off Experiment**

Naïve Huh7 cells and Huh7 cells (1x10^6^) infected with DENV at a MOI of 10 TCID_50_ per cell for 24 h were treated with 100 µg/ml harringtonine or DMSO for 1.5, 3 or 4.5 min prior to CHX treatment and cell lysis. Cell extracts were processed as described above for polysome profile analysis.

**Cell Lysis and Immunoblotting**

For Western blot analysis 1x10^5^ Huh7 cells were lysed with 100 µl ice cold protein lysis buffer (50 mM Tris-HCl pH7.4, 150 mM NaCl, 15 mM NaCl_2_, 1% Triton X-100) supplemented with EDTA-free protease inhibitor cocktail (Roche) and phosphatase inhibitors (60 mM β-glycerophosphate, 15 mM 4-nitrophenylphosphate, 1 mM sodium orthovanadate, 1 mM sodium fluoride) for 30 min on ice. Samples were centrifuged for 30 min at 13,000 rpm at 4°C and supernatants stored at -20°C. Total protein concentrations were measured by Bradford assay. Fifty µg total proteins were denatured in 6x Laemmli sample buffer, separated by SDS-PAGE and transferred to a PVDF membrane. Membranes were blocked by overnight incubation at 4°C in Tris-buffered saline containing 0.01% Tween 20 (Sigma-Aldrich) and 5% milk or 5% BSA. Immunostaining was performed in the same buffer using appropriate first and secondary antibodies. Proteins were detected by using the ECL Plus Western Blotting Detection System (Pierce, GE Healthcare) according to the instructions of the manufacturer. Signal was detected using the Advance ECL Chemocam Imager (Intas Science Imaging) and band quantified using LabImage 1D Software (Intas Science Imaging).

The following primary antibodies and corresponding blocking buffers were used: mouse monoclonal anti-eIF2α (Cell Signaling; BSA; 1:1000), rabbit polyclonal anti-phospho-eIF2α (Cell signaling; BSA; 1:500), rabbit polyclonal anti-PKR (Santa Cruz; milk; 1:500), rabbit polyclonal anti-phospho-PKR (Epitomics; BSA; 1:500), rabbit polyclonal anti-eIF4E (Cell Signaling; BSA; 1:2000), rabbit polyclonal anti-phospho eIF4E Ser209 (Cell Signaling; BSA; 1:500), monoclonal rabbit anti-4EBP1 (Cell Signaling; BSA; 1:1000), rabbit polyclonal anti-p38α (SantaCruz; BSA; 1:1000), rabbit monoclonal anti-phospho-p38α Thr180/Tyr182 (Cell Signaling; BSA; 1:1000), rabbit monoclonal anti-Mnk1 (Cell Signaling; BSA; 1:1000), rabbit polyclonal anti-phospho Mnk1 (Cell Signaling; BSA; 1:500), mouse monoclonal anti-puromycin (Millipore; milk; 1:1000), goat polyclonal anti-eIF3B (Santa Cruz; BSA; 1:500), mouse monoclonal anti-eIF3E (Santa Cruz; BSA; 1:500), polyclonal rabbit anti-eIF4G (Cell Signaling; BSA; 1:1000), goat polyclonal anti-eIF4A (Santa Cruz; BSA; 1:500), rabbit polyclonal anti-eEF2 (Cell Signaling; BSA; 1:1000), polyclonal rabbit anti-phospho-eEF2 (Cell Signaling; BSA; 1:1000), mouse monoclonal anti-PABP (Santa Cruz; BSA; 1:500), mouse monoclonal anti-GAPDH (Santa Cruz; milk; 1:5,000), mouse monoclonal anti-β-actin (Sigma-Aldrich; milk; 1:5000), monoclonal mouse anti-HA (Sigma-Aldrich; milk; 1:3000).

DENV proteins were detected by using the following antisera: rabbit polyclonal anti-NS3 (10), rabbit polyclonal anti-NS4B (10). Of note, the polyclonal anti-DENV NS3 cross-reacted with ZIKV MR766 and WNV NY NS3 proteins.

**Phos-tag Gel Analysis**

Phos-tag analysis as described by Kinoshita and colleagues (15) was performed to detect mobility shift of phosphorylated eIF2α and eIF4E. A resolving gel was casted containing 70 µM Phos-tag acrylamide and 140 µM Mn^2+^ as recommended by the manufacturer (Wako Pure Chemical Industries). Before blotting, SDS-PAGE was incubated for 10 min in transfer buffer (25 mM Tris-HCl, pH 8.3, 150 mM Glycine, 20 % methanol) supplemented with 1mM EDTA, followed by incubation for 10 min in transfer buffer.

**m^7^GTP Immuno-precipitation**

Naïve Huh7 cells (3x10^6^), Huh7 cells infected with DENV for 24 h and Huh7 cells treated with 2.5 µM Torin1 (Tocris) for 16 h were lysed in immunoprecipitation lysis buffer (50 mM Tris-HCl pH 7.8, 150 mM NaCl, 1 mM EDTA, supplemented with EDTA-free protease inhibitor cocktail) for 40 min on ice and centrifuged at 13,000 rpm for 20 min at 4°C. Ten percent of total cell extracts were kept for analysis of the input fraction. Rest cell extract was incubated with 50 µl of ɣ-Aminophenyl-m^7^GTP Agarose C_10_-linked beads (Jena Biosciences) and tumbled at 4°C for 6 h. Beads were washed 5 times by addition of 500 µl wash buffer (10 mM Tris-HCl pH 7.8, 150 mM NaCl, 0,1% NP40, supplemented with EDTA-free protease inhibitor cocktail) and centrifugation for 3 min at 5,000 rpm and 4°C. Samples were eluted in 100 µl 1x Laemmli buffer by incubation for 10 min at 95°C. Twenty-five percent of the eluate (25 µl) as well as 1% total extract (input) were loaded on gel for Western blot analysis. Signals were detected and quantified as described above. Values were normalized to the eIF4E signal intensity and relative to naïve cells (Mock).

**Immunofluorescence Microscopy and Antibodies**

For fluorescence immunostaining cells were fixed for 15 min with 4% (w/v) paraformaldehyde in PBS, permeabilized by 5 min-treatment with PBS containing 0.5% Triton X-100 and incubated in blocking buffer (5% goat serum, 5% sucrose in PBS) for 30 min. Cells were sequentially incubated with primary and secondary antibodies diluted in blocking buffer. For SG-specific staining, cells were further permeabilized with 100% ice-cold methanol for 10 min at -20°C prior to treatment as described above. Cells were stained with goat polyclonal anti-eIF3B (Santa Cruz; 1:500), mouse monoclonal anti-HuR (3A2) (Santa Cruz; 1:500), mouse monoclonal anti-PCBP2 (Abnova; 1:500), goat polyclonal anti-TIA-1 (Santa Cruz, 1:500).

DENV proteins were detected by using the following antisera: immuno-purified rabbit polyclonal anti-NS1 ((16), 1:50-1:100), immuno-purified rabbit polyclonal anti-NS3 ((10), 1:200), rabbit polyclonal anti-NS4B (GeneTex, 1:500), rabbit polyclonal anti-NS5 (GeneTex, 1:1000), rabbit polyclonal anti-Capsid (GeneTex, 1:1000), immuno-purified mouse monoclonal anti-Envelope (3H5-1, 1:250). ZIKV MR766 and WNV NY infection were visualized by staining dsRNA using the mouse monoclonal J2 antibody (Scicons; 1:400). Protease 3C of HAV was detected using the rabbit polyclonal anti-3C antibody kindly provided by Yuri Kusov (University of Lübeck, Germany). Of note, cells were permeabilized with 50 µg/ml digitonine in PBS for 15 min instead of Triton-X100 treatment. Coverslips were mounted by using Fluoromount G Reagent (Southern Biotech). Fluorescence images were acquired with a Nikon Ti Eclipse microscope and fluorescence signals were analyzed with the NIS-Element AR software package. Confocal images were acquired with a Leica TCS SP5 and fluorescence signals were analyzed with the LAS AF software. All images were processed using the ImageJ software package Fiji (http://fiji.sc/wiki/index.php/Fiji) (17).

**Polyprotein Transfection**

Huh7 Lunet T7 cells (1,5x10^5^) were seeded in a 6-well plate and transfected with Effectene R (Qiagen) according to manufacturer instructions. In brief, medium was replaced 1 h before transfection. One µg of pSM-NS1-5 (DENV NGC) or pTM HAV was mixed with 8 µl Enhancer Solution and diluted to a final volume of 100 µl in EC buffer. After 5 min incubation at room temperature, 10 µl Effectene R reagent was added and the mix was incubated for 15 min at room temperature. Medium was added to the DNA mix and added dropwise on the cells. Medium was replaced 8 h post transfection.

**Ribopuromycylation Assay and Quantification of Fluorescence Intensities**

*De novo* synthesized proteins were quantified by measuring the incorporation of puromycin on native peptide chains as described previously (18, 19). For fluorescence analysis, Huh7 (1x10^5^) cells were infected with DENV NGC, ZIKV MR766 and WNV NY at an MOI of 0.5 TCID_50_ or pfu per cell for 24, 36 and 48 h. Shortly before harvesting, control cells were treated with 0.5 mM arsenite to induce SGs and inhibit host cell translation. Cells were incubated for 5 min at 37°C with 10 µg/ml puromycin (Gibco, Life Technologies), washed twice with PBS, fixed with 4% paraformaldehyde in PBS for 15 min at room temperature and permeabilized with 0.5% Triton X-100 in PBS for 5 min. Cell were further stained with mouse monoclonal anti-puromycin (Millipore; 1:1000) to visualized released puromycylated chains. Coverslips were mounted as described above. Fluorescence intensities were quantified by using the ImageJ software package. In brief, a region of interest (ROI) was drawn around individual naïve cells or infected cells, as determined according to the NS3 signal (green) and intensity of the puromycin (red) signal was measured for each ROI using the ImageJ software package Fiji (17).

**Induction of Stress Granules by Drug Treatment and Image Analysis**

Huh7 cells (1x10^5^) were seeded into a 6-well plate containing 4 coverslips. Cells were infected with DENV NGC, ZIVK MR766 and WNV NY at a MOI of 0.5 TCID_50_ or pfu per cell for 24, 36 and 48 h and subsequently treated or not with drugs inducing SGs. After treatment, cells were fixed for immunostaining. Oxidative stress was induced by treating cells with 500 µM Na-Arsenite (Sigma-Aldrich) contained in culture medium for 45 min at 37°C. Metabolic stress was induced by incubation of cells with 10 µM carbonyl cyanide 4-(trifluoromethoxy)phenyl­hydrazone (FCCP) (Sigma-Aldrich) in glucose-free DMEM for 1 h. ER-stress was induced by incubation of cells with DMEM containing 10 µM thapsigargin (Biotrends) for 1 h at 37°C. eIF2α-independent SGs were induced 30 h post infection by treatment with 1 µM hippuristanol for 8 h. After treatment, cells were harvested for further analysis or fixed for immunostaining.

SG numbers were quantified using the plugin “Analyze Particle” of the ImageJ software package Fiji (17). A constant threshold was set for each experiment according to naïve control cells to allow particle quantification in individual ROIs (particle size: 0.5-20 µm²). NS5 signal intensity of infected cells was measured using as described above for the puromycin signal intensity.

**Phospho-antibody Array**

The proteome Profiler Human Phospho-MAPK Array (R&D Systems) was used to analyze activation of p38 and ERK isoforms using 300 µg of Huh7 cell lysates of Mock-, UV-inactivated- and DENV-infected cells harvested at 24 and 48 h post infection, according to the manufacturer´s instructions. The signal was detected on radiographic film (Fuji RX) and quantified using the Fiji software package (17).

**Cytotoxicity Measurement**

Huh7 cells (1x10^4^) were seeded in triplicate wells into 96-well plates. Cells were treated for 16 h with CGP57380 (Mnk1 inhibitor, Tocris) or SB203580 (p38 Inhibitor, Sigma-Aldrich) or with DMSO as control. Cell viability was determined by quantitation of ATP using the CellTiter-Glo assay (Promega) as recommended by the manufacturer. Fluorescence was measured at 560 nm using a Mithras LB 940 plate reader (Berthold Industries). Incubation with DMSO solvent and untreated cells served as control.

**Estimation of DENV Titers upon Inhibitor Treatment**

For CGP57380 treatment, Huh7 cells (1x10^5^) were infected with DENV or ZIKV MR766 at a MOI of 0.1 TCID_50_ or pfu per cell for 8 h before addition of 50 µM CGP57380 for 16 h. For SB203580 treatment, cells were simultaneously infected and treated with 50 µM SB203580 for 24 h. Supernatants were harvested, filtered through 0.45 µM membrane and titrated by limiting dilution assay or plaque assay.

**Statistical Significance**

Statistical analysis was performed by using the GraphPad Prism software (GraphPad). Statistical significance was calculated by performing a two-tailed Student’s t test or one sample t-test (***, p < 0.001; **, p < 0.01; *, p < 0.05).

**Supplemental References**

1. **Ruggieri A, Dazert E, Metz P, Hofmann S, Bergeest JP, Mazur J, Bankhead P, Hiet MS, Kallis S, Alvisi G, Samuel CE, Lohmann V, Kaderali L, Rohr K, Frese M, Stoecklin G, Bartenschlager R.** 2012. Dynamic oscillation of translation and stress granule formation mark the cellular response to virus infection. Cell HostMicrobe **12:**71-85.

2. **Backes P, Quinkert D, Reiss S, Binder M, Zayas M, Rescher U, Gerke V, Bartenschlager R, Lohmann V.** 2010. Role of annexin A2 in the production of infectious hepatitis C virus particles. J Virol **84:**5775-5789.

3. **Gualano RC, Pryor MJ, Cauchi MR, Wright PJ, Davidson AD.** 1998. Identification of a major determinant of mouse neurovirulence of dengue virus type 2 using stably cloned genomic-length cDNA. J Gen Virol **79 ( Pt 3):**437-446.

4. **Pryor MJ, Carr JM, Hocking H, Davidson AD, Li P, Wright PJ.** 2001. Replication of dengue virus type 2 in human monocyte-derived macrophages: comparisons of isolates and recombinant viruses with substitutions at amino acid 390 in the envelope glycoprotein. Am J Trop Med Hyg **65:**427-434.

5. **Kumar A, Buhler S, Selisko B, Davidson A, Mulder K, Canard B, Miller S, Bartenschlager R.** 2013. Nuclear localization of dengue virus nonstructural protein 5 does not strictly correlate with efficient viral RNA replication and inhibition of type I interferon signaling. J Virol **87:**4545-4557.

6. **Gauss-Muller V, Kusov YY.** 2002. Replication of a hepatitis A virus replicon detected by genetic recombination in vivo. J Gen Virol **83:**2183-2192.

7. **Ran FA, Hsu PD, Wright J, Agarwala V, Scott DA, Zhang F.** 2013. Genome engineering using the CRISPR-Cas9 system. Nat Protoc **8:**2281-2308.

8. **Heigwer F, Kerr G, Boutros M.** 2014. E-CRISP: fast CRISPR target site identification. Nat Methods **11:**122-123.

9. **Sanjana NE, Shalem O, Zhang F.** 2014. Improved vectors and genome-wide libraries for CRISPR screening. Nat Methods **11:**783-784.

10. **Miller S, Sparacio S, Bartenschlager R.** 2006. Subcellular localization and membrane topology of the Dengue virus type 2 Non-structural protein 4B. J Biol Chem **281:**8854-8863.

11. **van den Hoff MJ, Christoffels VM, Labruyere WT, Moorman AF, Lamers WH.** 1995. Electrotransfection with "intracellular" buffer. Methods MolBiol **48:**185-197.

12. **Lindenbach BD, Evans MJ, Syder AJ, Wolk B, Tellinghuisen TL, Liu CC, Maruyama T, Hynes RO, Burton DR, McKeating JA, Rice CM.** 2005. Complete replication of hepatitis C virus in cell culture. Science **309:**623-626.

13. **Hofmann S, Cherkasova V, Bankhead P, Bukau B, Stoecklin G.** 2012. Translation suppression promotes stress granule formation and cell survival in response to cold shock. MolBiolCell **23:**3786-3800.

14. **Livak KJ, Schmittgen TD.** 2001. Analysis of relative gene expression data using real-time quantitative PCR and the 2(-Delta Delta C(T)) Method. Methods **25:**402-408.

15. **Kinoshita E, Kinoshita-Kikuta E, Takiyama K, Koike T.** 2006. Phosphate-binding tag, a new tool to visualize phosphorylated proteins. Mol Cell Proteomics **5:**749-757.

16. **Welsch S, Miller S, Romero-Brey I, Merz A, Bleck CK, Walther P, Fuller SD, Antony C, Krijnse-Locker J, Bartenschlager R.** 2009. Composition and three-dimensional architecture of the dengue virus replication and assembly sites. Cell Host Microbe **5:**365-375.

17. **Schindelin J, Arganda-Carreras I, Frise E, Kaynig V, Longair M, Pietzsch T, Preibisch S, Rueden C, Saalfeld S, Schmid B, Tinevez JY, White DJ, Hartenstein V, Eliceiri K, Tomancak P, Cardona A.** 2012. Fiji: an open-source platform for biological-image analysis. NatMethods **9:**676-682.

18. **David A, Dolan BP, Hickman HD, Knowlton JJ, Clavarino G, Pierre P, Bennink JR, Yewdell JW.** 2012. Nuclear translation visualized by ribosome-bound nascent chain puromycylation. JCell Biol **197:**45-57.

19. **Panas MD, Kedersha N, McInerney GM.** 2015. Methods for the characterization of stress granules in virus infected cells. Methods.
